# Developing Highly Effective Nanoparticle mRNA Therapeutic for Pediatric Acute Respiratory Distress Syndrome

**DOI:** 10.1002/advs.77813

**Published:** 2026-09-17

**Authors:** Zicheng Deng, Wen Gao, Jonathan Do, Danli Lu, Ying‐Wei Lan, Andreas Damianos, Gautam Verma, Ali Al Siraj, Isabella Lowry, Lance Peter, Nicholas E. Banovich, Fei Sun, Hua He, Tanya V. Kalin, Vladimir V. Kalinichenko

**Affiliations:** ^1^ Phoenix Children's Research Institute, Department of Child Health University of Arizona College of Medicine Phoenix United State; ^2^ Division of Neonatology Phoenix Children's Hospital Phoenix United State; ^3^ Division of Bioinnovation and Genome Sciences Translational Genomics Research Institute United State; ^4^ Key Laboratory of Birth Defects and Related Diseases of Women and Children of MOE West China Second University Hospital Sichuan University Chengdu Sichuan China; ^5^ NHC Key Laboratory of Chronobiology Sichuan University Chengdu Sichuan China; ^6^ The Joint Laboratory for Lung Development and Related Diseases of West China Second University Hospital Sichuan University and School of Life Sciences of Fudan University Chengdu Sichuan China; ^7^ Phoenix Children's Center for Cancer and Blood Disorders Phoenix United State

**Keywords:** endothelial cells, gene therapy, nanoparticle delivery system, pediatric acute respiratory distress syndrome, sepsis

## Abstract

Sepsis‐induced pediatric acute lung injury (ALI) and pediatric acute respiratory distress syndrome (PARDS) are life‐threatening conditions with high mortality rates and no current cure. Most ALI/ARDS studies focus on adults, albeit the pediatric population has unique challenges often underrepresented. ALI/PARDS severely impacts pulmonary endothelial cells (ECs), causing endothelial dysfunction and vascular leakage. FOXF1 is a transcription factor critical for lung repair after injury, representing a viable target for ALI/PARDS. This study developed and tested a novel nanoparticle system for precise delivery of *FOXF1* mRNA into lung ECs to reduce endothelial damage and improve lung function in mouse model of PARDS. Systemic inflammatory response was induced in neonatal mice after intraperitoneal administration of lipopolysaccharide (LPS). Specifically designed nanoparticles (NP) were used to intravenously deliver stabilized *FOXF1* mRNA (*FOXF1* NP) after LPS injury to restore FOXF1 expression in injured lung endothelial cells. *FOXF1* NP selectively targeted pulmonary ECs without affecting other cell types or organs. *FOXF1* NP treatment reduced vascular leakage, enhanced endothelial barrier function, and improved survival of neonatal mice after injury. *FOXF1* NP decreased EC apoptosis by restoring the expression of BCL2, an anti‐apoptotic FOXF1 target gene. Nanoparticle‐based rescue of lung ECs has promise for future treatments of human ALI/PARDS.

## Graphical abstract

Sepsis‑induced pediatric acute respiratory distress syndrome suppresses FOXF1 in lung endothelial cells which causes life‐threatening lung damage. To counter this, researchers developed nanoparticles that specifically target these cells and deliver *FOXF1* mRNA. The treatment protects the endothelium, prevents cell apoptosis by restoring the BCL2 gene, and improves survival of mice after severe lung injury.

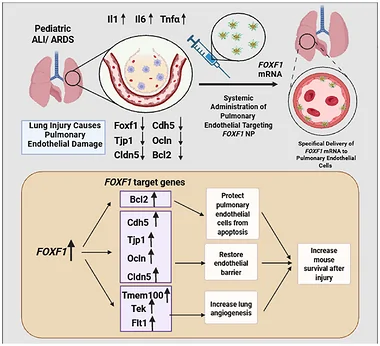

## 1 Introduction

Pediatric acute respiratory distress syndrome (PARDS), a life‐threatening complication of acute lung injury (ALI) in children, remains a major cause of morbidity and mortality [1,2]. Mortality rates are significantly higher in children who develop ARDS following critical injury (34.0%) than those who do not (1.7%) [3]. In severe cases, mortality can reach 50% [4]. Epidemiological studies highlight differences in ARDS prevalence and outcomes between children and adults, particularly in neonates [5,6]. These temporal disparities due to age are considered to arise from the unique biological characteristics of PARDS, influenced by the level of the lung developmental stage, chest wall mechanics, and unique cellular responses to inflammation, apoptosis, and tissue repair mechanisms [7,8]. This recognition led to the development of specific clinical criteria for the diagnosis of PARDS (PALICC). Endothelial cell (EC) dysfunction is critical in the pathogenesis of ALI/PARDS [9]. Inflammatory‐induced EC dysfunction compromises microvascular barrier integrity, leading to vascular leakage into the interstitium and subsequently into the alveolar space resulting in alveolar and vascular damage with potential micro‐thrombosis. These findings lead to ventilation to perfusion imbalances seen in the acute phase of the syndrome which directly affect the ability to oxygenate resulting in acute respiratory failure and are drivers of the high morbidity and mortality in ALI/ARDS. Excessive endothelial loss as well as inefficient resolution of the inflammatory response results in alveolar loss and vascular remodeling which can persist, contributing to the long‐term complications of ALI/PARDS [10]. Current interventions mainly support the symptomatology to ensure survival but not the underlying pathophysiology [1,8]. Despite advances in pediatric care, no effective treatment specifically targets endothelial dysfunction [1,11], representing a critical unmet need.

Gene therapy holds promise for restoring EC function by targeting key molecular pathways. FOXF1, a transcription factor crucial for endothelial integrity, angiogenesis, and lung repair [12, 13, 14, 15, 16, 17], has emerged as a potential therapeutic target. FOXF1 regulates the expression of Vascular Endothelial Growth Factor (VEGF) receptor 2 and VEGF signaling in pulmonary ECs [18]. Loss of *Foxf1* in ECs leads to lung inflammation, edema, and death in mice [19], supporting a potential gene therapy approach to stimulate FOXF1 function after lung injury. However, gene therapy faces challenges due to the limitations of viral vectors, which pose safety concerns such as immunogenicity, cargo limitations, and insertional mutagenesis [20, 21, 22, 23].

Nanoparticle (NP)‐based delivery systems offer a promising alternative solution to viral vectors [24, 25, 26, 27]. Poly (β‐amino ester) (PBAE), a biodegradable polymer, enables biocompatible and tunable nucleic acid delivery [28, 29, 30]. As a cationic polymer, PBAE naturally forms positively charged NPs, facilitating interaction with the negatively charged endothelial glycocalyx via electrostatic attraction [31, 32, 33]. Recently, we developed lung specific PBAE NPs capable of delivering plasmid DNAs to pulmonary ECs in adult mice [34]. The chemical synthesis and functionalization of these PBAE polymers are illustrated in Figure S1. However, their therapeutic potential in delivering mRNAs and targeting efficiency in lung diseases remain unclear.

**FIGURE 1:**
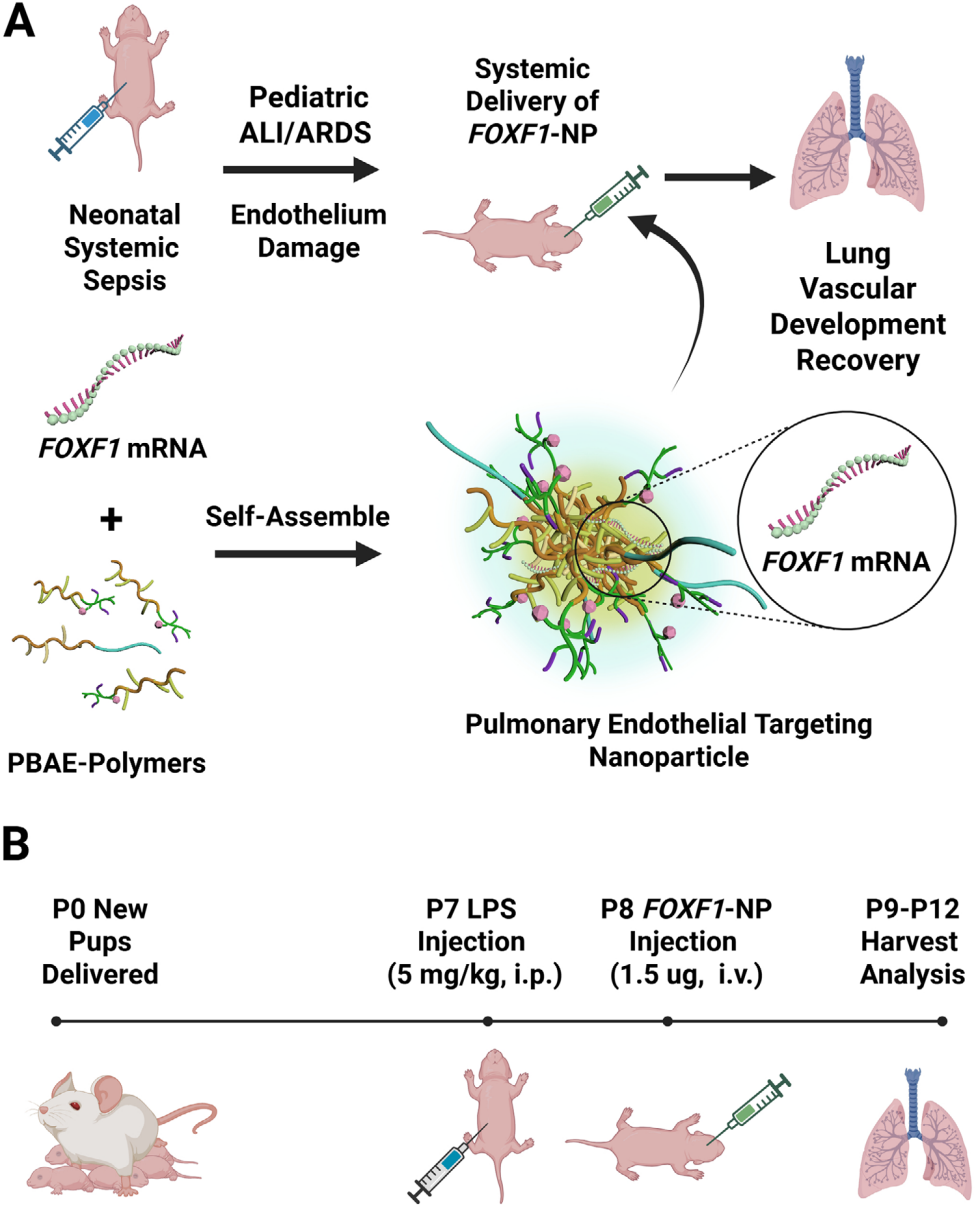
Experimental design for NP mediated *FOXF1* mRNA delivery in a mouse model of pediatric ALI/ARDS. (A) Schematic overview of the study: Pediatric ALI/ARDS was induced via LPS induced systemic sepsis, leading to endothelial damage. *FOXF1* NPs were delivered intravenously to target lung endothelial cells. (B) Timeline: LPS (5 mg/kg, i.p.) was administered on P7, followed by *FOXF1* NP delivery (1.5 µg, i.v.) on P8. Tissues were harvested for analysis between P9 and P12. Created with BioRender.com.

Here, we established an ALI/PARDS model using systemic sepsis induced by lipopolysaccharide (LPS), because non‐pulmonary sepsis is the second most common risk factor for PARDS [1,2]. We demonstrated that endothelial FOXF1 expression is dramatically decreased after septic lung injury. We next tested whether restoration of FOXF1 levels will improve the PARDS phenotype. We developed a PBAE‐based mRNA delivery system that efficiently and specifically targets pulmonary ECs and delivers *FOXF1* mRNA in mice, protecting ECs from apoptosis, improving lung function, and increasing mice survival after ALI/PARDS (Figure 1).

## 2 Results

### 2.1 LPS Administration Induces Inflammatory Response Detectable in BALF

Pro‐inflammatory cytokines *Il1*, *Il6*, and *Tnfα* were assessed via qRT‐PCR in BALF cell specimens collected 30 min after intraperitoneal LPS injection. Their expression was found significantly increased in the LPS‐administered group compared to controls (Figure S2). These findings demonstrate that systemic LPS administration to neonates induces a rapid inflammatory response detectable in BALF. One day post‐injection, body weights were found significantly reduced in LPS‐injured mice compared to controls (Figure S3A). BALF cell analysis 16 h after LPS injection revealed a marked increase in the number of immune cells (Figure S3B), with cytospin preparations showing abundant macrophages (Figure S3C). Macrophages and neutrophils in BALF were identified using flow cytometry with simultaneous detection of CD45, CD11b, SiglecF, Ly6G, Ly6C, and F4/80 (Figure S4). Total cell and macrophage numbers were significantly increased in the LPS‐administered group compared to controls (Figure S5A‐B). In contrast, neutrophil counts in BALF of neonatal mice remained unchanged, consistent with previous studies of neonatal lung injury [35,36].

### 2.2 LPS Administration Induces Lung Tissue Inflammation, EC Loss and EC Barrier Disruption

**FIGURE 2:**
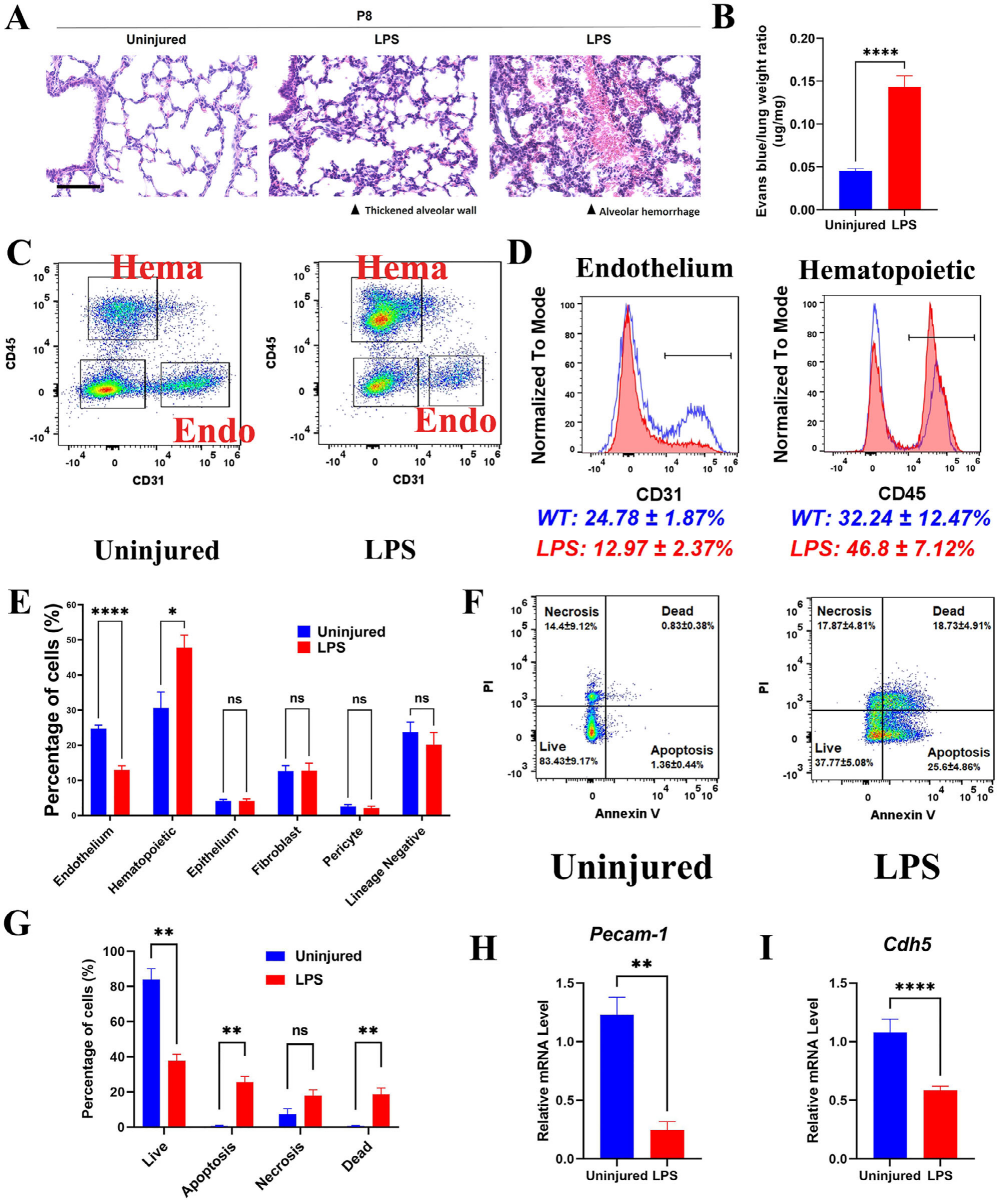
LPS‐induced pulmonary EC damage in mouse model of PARDS. (A) Representative H&E‐stained lung sections show thickened alveolar walls and alveolar hemorrhage in lungs of LPS‐treated neonatal mice. Scale bar is 50 µm, (B) Vascular permeability analysis using Evans blue dye shows increased pulmonary leakage after LPS treatment. (C) Representative flow cytometry plots visualize endothelial and hematopoietic cell populations in uninjured and LPS‐injured neonatal mouse lungs. (D) Quantification of endothelial and hematopoietic cell populations is performed using histograms obtained by flow cytometry. (E) Bar graphs show the percentages of endothelial cells, hematopoietic cells, epithelial cells, fibroblasts, pericytes, and lineage‐negative cells in lung tissue with or without sepsis. (F) Flow cytometry analysis of pulmonary EC apoptosis was performed using Annexin V/PI staining. (G) Quantification of live, apoptotic, necrotic, and dead cell populations is shown with bar graphs. (H‐I) qRT‐PCR analysis shows significantly decreased expression of *Pecam‐1* and *Cdh5* mRNAs in LPS‐treated lungs. Data are presented as mean ± SEM (n = 5). Statistical significance: *p ≤ 0.05, **p ≤ 0.01, ***p ≤ 0.001, ****p ≤ 0.0001, ns is not significant.

We next evaluated the expression of *Il1*, *Il6*, and *Tnfα* via qRT‐PCR in total lung RNA in order to assess the presence of inflammatory response in the lung tissue (Figure S6). The cytokine expression was markedly increased after LPS injury (Figure S6) mirroring the findings from the BALF specimens (Figure S2). Lung tissue histology revealed thickened alveolar walls and alveolar hemorrhage in LPS‐treated mice (Figure 2A), both pathognomonic features of ALI/PARDS [37, 38]. Vascular permeability assay using Evans blue dye showed significant endothelial leakage following LPS exposure (Figure 2B). A marked decrease in EC numbers was noted, via flow cytometry (Figure S7), one day after LPS injection (Figure 2C–E) with increase in the apoptotic and dead endothelial cells (Figure 2F‐G), indicating severe EC injury. This was further supported by the downregulation of endothelial markers Pecam‐1 and Cdh5 (VE‐cadherin) in the LPS‐administered group compared to controls (Figure 2H‐I). Expression of genes involved in the tight junction function of ECs such as *Tjp1* (ZO‐1) and *Ocln* (Occludin) was found also decreased after LPS injection (Figure S8), consistent with disruption of EC barrier. Of note, analysis of the liver and kidney revealed marked leukocytosis confirming the systemic inflammatory response induced by LPS administration (Figure S9A‐B). The EC numbers were not found significantly decreased in the liver, but in the kidney EC numbers were reduced (Figure S9A–B). Thus, LPS administration in neonatal mice induces systemic inflammatory response leading to pulmonary and kidney EC loss, increased vascular permeability and EC apoptosis in the lung tissue, and systemic leukocytosis, all hallmarks of sepsis and PARDS.

### 2.3 Nanoparticle Properties and Pulmonary EC Targeting in Neonatal Mice with ALI/PARDS

**FIGURE 3:**
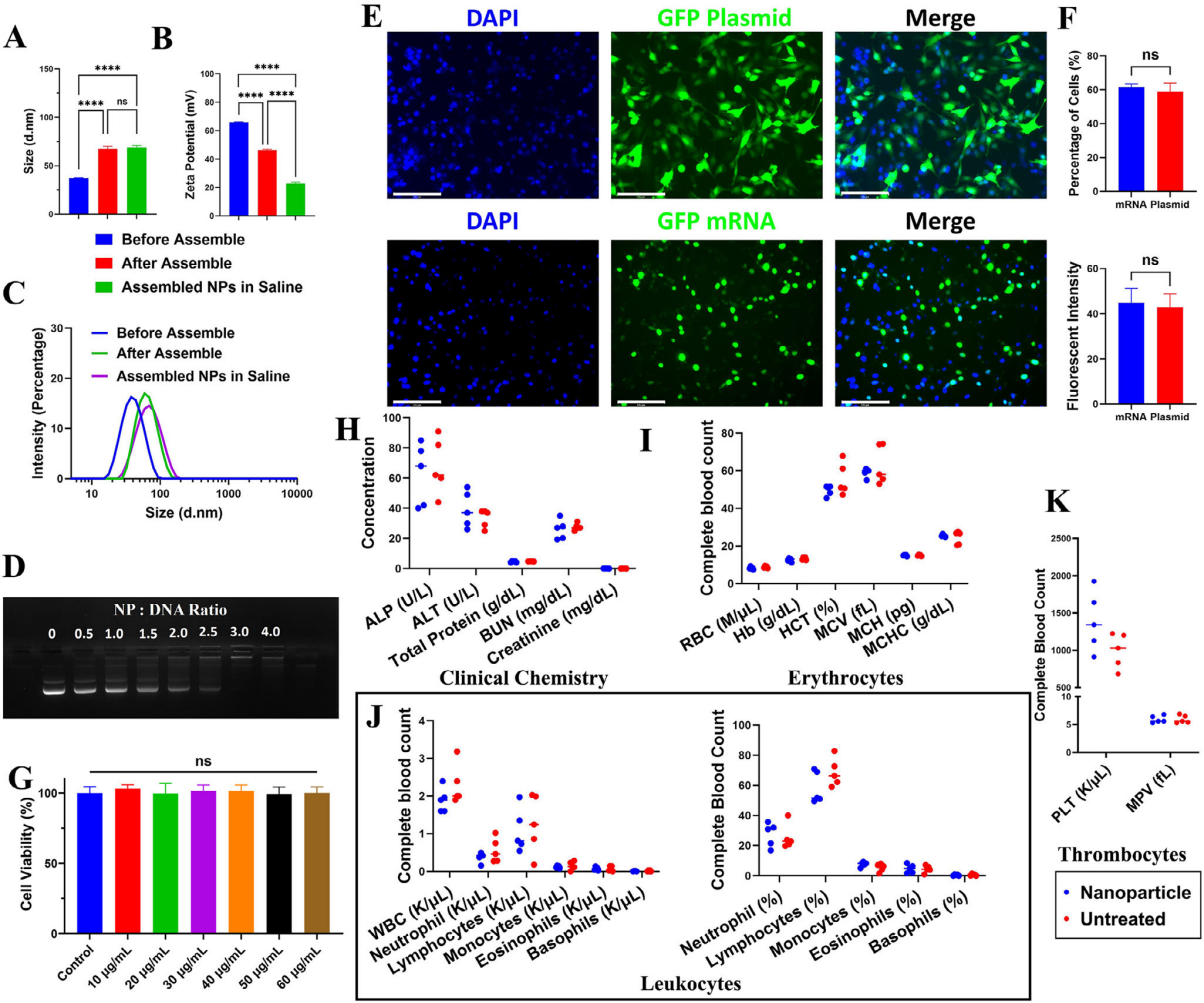
Characterization, in vitro transfection efficiency, and safety profile of PBAE NPs. (A, B) NP size and zeta potential before and after assembly with luciferase mRNA, and the assembled NPs in saline. (C) Size distribution of the NPs before and after assembly, and the assembled NPs in saline. (D) Gel electrophoresis shows mRNA encapsulation in NPs with different nanoparticle: mRNA ratios. (E) Representative images show MFLM‐91U ECs transfected with NPs containing GFP reporter plasmid DNA and GFP mRNA. Nuclei were counterstained with DAPI (blue). Scale bars are 75 µm. (F) Quantification of transfection efficiency and fluorescence intensity was performed using fluorescence images. (G) Cell viability profiles of transfected cells after treatment with different concentrations of nanoparticles. (H‐K) Clinical chemistry and hematological analyses were performed using peripheral blood from NP‐injected and untreated mice for NP toxicity study. Data are presented as mean ± SEM (n = 5). Statistical significance: *p ≤ 0.05, **p ≤ 0.01, ***p ≤ 0.001, ****p ≤ 0.0001, ns is not significant. Abbreviations: Alkaline phosphatase (ALP), Alanine aminotransferase (ALT), Blood urea nitrogen (BUN), Red blood cell (RBC), Hemoglobin (Hb), Hematocrit (HCT), Mean corpuscular volume (MCV), Mean corpuscular hemoglobin (MCH), Mean corpuscular hemoglobin concentration (MCHC), White blood cell (WBC), Platelet count (PLT), Mean platelet volume (MPV).

Since the pulmonary endothelium is significantly affected in ALI/PARDS, we sought to target this cell type using the previously developed NP delivery system, which selectively targets lung ECs in the lung‐specific manner [34]. Using dynamic light scattering system (DLS) we assessed the physicochemical properties of the NPs. After assembly, the NP size significantly increased, while the surface charge (zeta potential) significantly decreased, confirming the self‐assembly process of NPs with mRNA (Figure 3A,B). The single peak in the size distribution indicates that the NPs maintain a relatively uniform size before and after assembly (Figure 3C). To evaluate the physicochemical properties of the NPs in physiological environments, we measured their size and surface charge in saline. The NP size showed no significant changes upon transition to saline, indicating excellent colloidal stability. Since previous studies on PBAE NPs indicate that nanoparticles with lower surface charges exhibit lower cytotoxicity, this reduction in charge is important to prevent toxicity [39]. While published studies showed that polyanionic nucleic acid nanostructures reduce cationic toxicity during transfection [40], a positive charge remains necessary for effective pulmonary EC targeting in vivo [31]. Therefore, the NP delivery system developed in this study balances safety with the requirements for endothelial‐specific targeting in vivo. To evaluate the batch‐to‐batch consistency and long‐term storage stability of the NPs, we characterized the polydispersity index (PDI) of samples synthesized in 6 different batches (Figure S10). The NPs were stored at ‐80°C for up to 5 months, and their PDI was measured both before and after the self‐assembly process. The PDI of the NPs remained low and stable in 6 district batches throughout the 5‐month storage period, indicating no significant aggregation during storage. These results show the batch‐to‐batch consistency and storage feasibility of the NPs for extended periods. Gel electrophoresis was used to determine the optimal polymer‐to‐mRNA mass ratio for NP stability, revealing that a complete nucleic acid encapsulation was achieved at 3:1 ratio (Figure 3D). To evaluate the transfection efficiency of NPs, mouse fetal lung endothelial (MFLM‐91U) cells were transfected in vitro with either GFP reporter plasmid DNA or GFP mRNA. Fluorescence imaging confirmed efficient transfection for both nucleic acid types (Figure 3E). Quantitative analysis showed that GFP mRNA exhibited similar transfection efficiency to plasmid DNA, and fluorescence intensity remained comparable between the two (Figure 3F). The CCK‐8 assay of transfected mouse MFLM‐91U ECs after treatment with different concentrations of nanoparticles showed no decrease in cell viability compared to the control group (Figure 3G). To evaluate the safety of NPs in vivo, mice received intravenous injections of the NPs and peripheral blood was collected for clinical chemistry and hematological analyses. No significant differences were observed between NP‑treated and untreated control groups. All parameters remained within normal physiological ranges (Figure 3H–K). Histopathological examination of major organs (heart, liver, spleen, lungs, and kidneys) revealed no morphological changes, indicating that NPs are biocompatible and do not induce systemic toxicity (Figure S11). To evaluate the systemic immune response following repeated administration, we performed a repeated injection experiment. NPs were administered every other day (48 h intervals) for a total of three doses. Blood samples were collected 30 min after the final injection for complement activation analysis. No significant changes in complement C5 were detected after three doses of NP injection compared to the uninjured group (Figure S12A–B), indicating that repeated NPs do not induce complement activation. To further assess the systemic inflammatory status, we collected mouse peripheral blood samples for immune cell counting and calculated the Systemic Inflammation Response Index (SIRI) (Figure S12C–D). Compared to the untreated group, three NP injections did not result in significant differences SIRI or blood cell counts.

**FIGURE 4:**
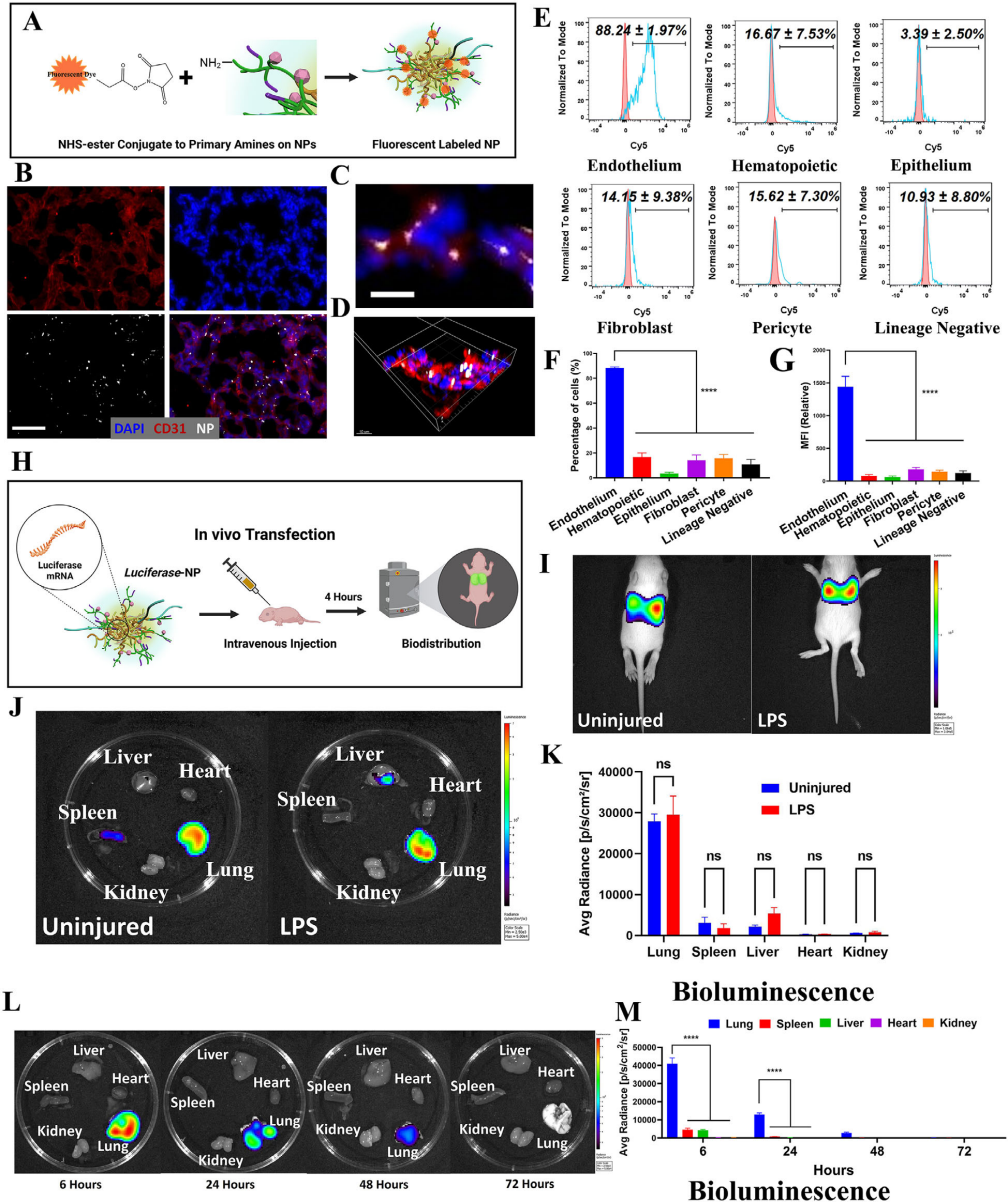
Pulmonary endothelial‐targeting efficiency of mRNA carried NPs injected via retro orbital route. (A) Fluorescent dye labeling of the nanoparticles. (B) Immunofluorescence imaging (Scale bar is 75 µm) of lung tissue sections stained with DAPI (blue) and CD31 (red) shows that NPs (white) are distributed throughout the pulmonary microvasculature. (C‐D) Immunofluorescence imaging (Scale bar is 15 µm) and 3D reconstruction (Scale bar is 10 µm) of lung tissue sections stained with DAPI (blue) and CD31 (red) shows that NPs (white) localized within pulmonary endothelial cells. (E) Flow cytometry analysis of Cy5‐labeled NPs lungs of neonatal mice injured with LPS. (F) Quantification of Cy5‐labeled NP targeting efficiency. (G) Mean fluorescence intensity (MFI) of Cy5‐labeled NPs. (H) Luciferase mRNA was encapsulated in NPs for the in vivo transfection and biodistribution study. Created with BioRender.com. (I) IVIS whole‐body imaging shows the in vivo bioluminescence after treatment with NPs carrying luciferase mRNA. (J) Luminescent images show the biodistribution of luciferase expression in dissected mouse organs after treatment with NPs carrying luciferase mRNA. (K) Quantification of luciferase signal intensity in different organs from uninjured and LPS‐injured mice. (L‐M) Representative IVIS images showing luciferase activity across major organs at 6, 24, 48, and 72 h post‐injection. Data are presented as mean ± SEM (n = 3‐5). Statistical significance: *p ≤ 0.05, **p ≤ 0.01, ***p ≤ 0.001, ****p ≤ 0.0001, ns is not significant.

For biodistribution studies in mice with ALI/PARDS, the NPs were labeled with the fluorescent dye Cy5 (Figure 4A). The Cy5 label enabled tracking of the NPs. The in vitro incubation confirmed that the NPs efficiently entered MFLM‐91U mouse ECs (Figure S13). To visualize NP localization in vivo, lung tissue sections were stained with CD31, demonstrating widespread NP distribution throughout the lung alveolar microvasculature, with strong co‐localization in ECs (Figure 4B–D). Flow cytometry analysis of lung tissue from injured P8 neonatal mice revealed that NPs selectively accumulated in pulmonary ECs, with minimal uptake by other lung cell populations (Figures 4E). Quantitative analysis confirmed the high targeting efficiency of NPs to ECs, with significantly lower NP uptake in hematopoietic cells, epithelial cells, fibroblasts, pericytes, and lineage‐negative cells populations (Figure 4F). Additionally, the mean fluorescence intensity (MFI) of Cy5‐labeled NPs was significantly higher in ECs, further confirming their preferential EC targeting (Figure 4G). NPs were also detected in endothelial cells of small pulmonary arteries and veins located in alveolar regions (Figure S14). To investigate the tissue distribution of NPs, the whole‐body bioluminescence imaging (IVIS) was performed to detect luciferase expression following NP‐mediated i.v. delivery of luciferase mRNA (Figure 4H). To determine the appropriate dosage for in vivo delivery, we performed a dose‐response study in neonatal mice (Figure S15). By delivering a luciferase reporter mRNA at doses ranging from 0.1 to 0.5 µg/g, we evaluated biodistribution and transfection efficacy via bioluminescence imaging. Bioluminescence intensity increased up to 0.3 µg/g dose and remained at similar levels with higher doses. Therefore, we selected 0.3 µg/g as the optimal dose for all subsequent experiments in this study. In both uninjured and LPS‐injured neonatal mice, luciferase expression was predominantly detected in the lungs with no significant difference between uninjured and LPS‐injured groups (Figure 4I–K). Thus, NPs with mRNA exhibit excellent pulmonary endothelial targeting efficiency, maintaining lung specificity after lung injury. We also evaluated the metabolic kinetic profile using a luciferase reporter mRNA. IVIS imaging was employed to monitor the systemic distribution and clearance rates across major organs (Figure 4L–M). The luciferase activity in the lung tissue was highest at 6 h post‐injection. The signal intensity decreased significantly by 24 h, indicating a rapid clearance from the lung tissue. By 72 h, the luciferase activity was undetectable (Figure 4L–M). No luciferase activity was observed in other organs at any time point. These results suggest that the mRNA delivery system exhibits high lung targeting efficiency. IVIS results and quantitative biodistribution analysis in a neonatal mouse model demonstrated that the nanoparticles exhibit high selectivity for the pulmonary microvasculature, with minimal accumulation in rapidly developing neonatal organs such as the liver, kidney, and spleen. By bypassing these high‐metabolic‐rate organs, the system reduces potential systemic toxicity while ensuring the therapeutic payload remains concentrated in pulmonary endothelial cells. To compare pulmonary delivery routes, we imaged the lung tissue following i.v. and intratracheal (i.t.) administration (Figure S16). The intravenous route showed a larger and more uniform distribution of nanoparticles in the lung, while intratracheal injection resulted in a lower luciferase activity in the lung tissue.

### 2.4 Loss of FOXF1 and Its Downstream Targets Across Pulmonary Endothelial Subtypes During Systemic Sepsis

**FIGURE 5:**
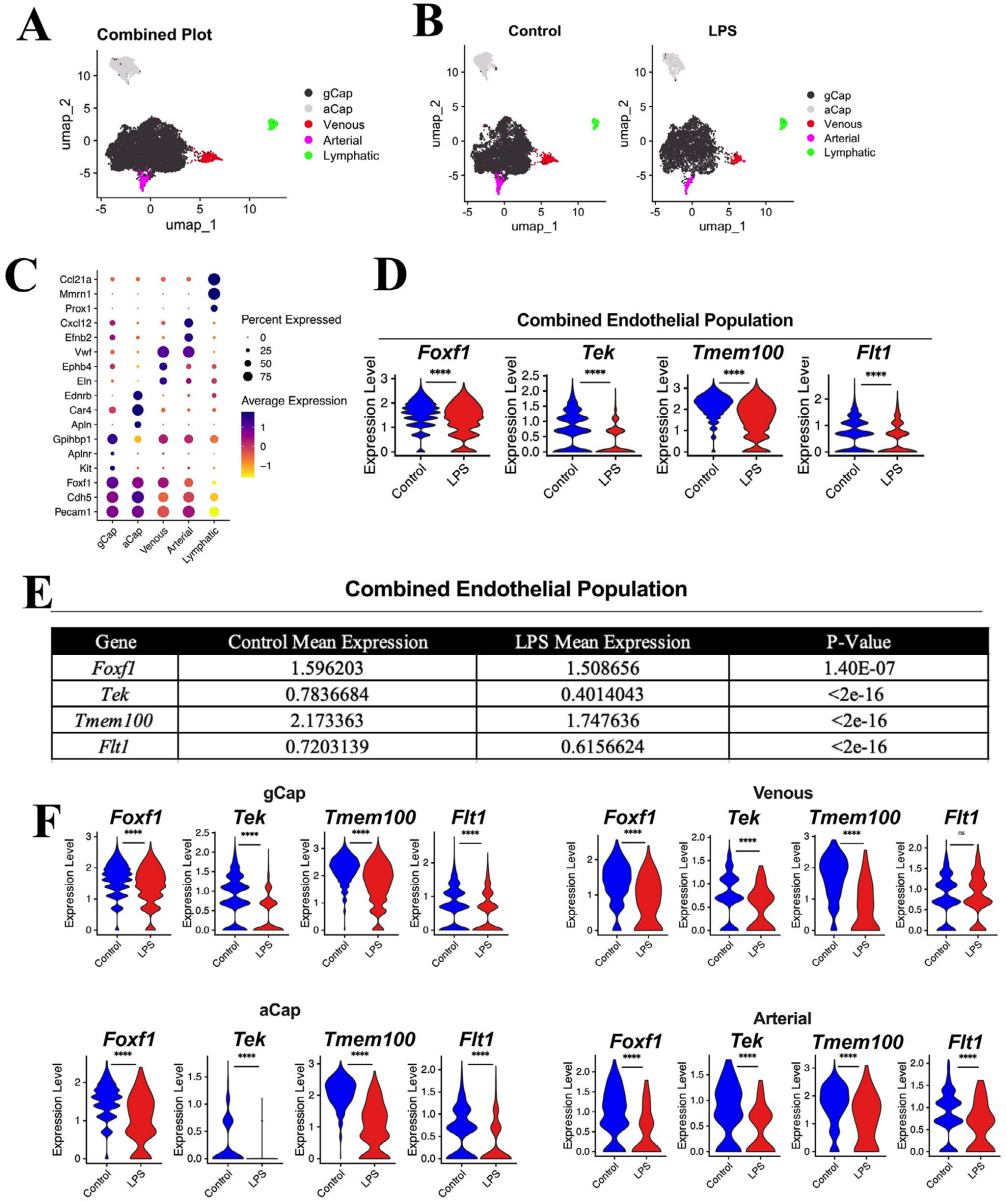
ScRNAseq analysis of FOXF1 downstream target genes in pulmonary endothelial cells 2 days after LPS induced lung injury. (A) Combined UMAP of the integrated control and LPS datasets shows five distinct endothelial cell populations expressing canonical cell‐specific markers. (B) Integrated UMAPs separated by treatment. Control (n = 8273 cells) and LPS (n = 4764). (C) Dotplot of various genes that were used to classify each pulmonary endothelial cell type. The size of the dot corresponds to the percentage of cells expressing the selected genes within the population while the darker color signifies a higher average expression. Conversely, the lighter shading represents a lower average expression. (D) Select genes known to be affected by LPS and regulated by FOXF1. (E) Average mean expression of total endothelial cells in control and LPS population of the genes depicted in D with calculated p‐values. Blue represents control group while red is LPS. (F) Select genes known to be affected by LPS and regulated by FOXF1 in different endothelial cell subtypes. Statistical significance: ****p ≤ 0.0001.

*FOXF1* expression is markedly reduced in pulmonary endothelial cells (ECs) following neonatal hyperoxic injury [41]. To determine whether this downregulation also occurs in a model of acute lung injury, we analyzed a publicly available single‐cell RNA‐sequencing (scRNA‐seq) dataset from control and LPS‐treated mice. Unsupervised clustering identified five transcriptionally distinct pulmonary EC subpopulations, general capillary (gCap), aerocyte capillary (aCap), arterial, venous, and lymphatic ECs (Figure 5A–B). Each EC subtype displayed a characteristic marker profile, as shown in the dot plot (Figure 5C), confirming accurate cluster annotation. We next explored the expression patterns of Foxf1 together with several well‑established downstream targets, including Tek (Tie2), Tmem100, and Flt1 (VEGFR1), all of which play essential roles in endothelial stability, vascular barrier integrity, and angiogenic recovery following injury. In the combined EC population, *Foxf1* expression was significantly reduced in LPS‐injured lungs, accompanied by concordant decreases in *Tek*, *Tmem100*, and *Flt1* expression (Figure 5D–E). This coordinated downregulation suggests that LPS‐induced sepsis disrupts Foxf1‐dependent transcriptional programs required for endothelial homeostasis and vascular repair. Subtype‐specific analyses revealed that LPS injury caused a broad and consistent suppression of Foxf1 and its target genes across all major EC subtypes, including gCap, aCap, arterial, and venous ECs (Figure 5F). These findings highlight that Foxf1 loss is not confined to a single vascular niche but instead represents a global endothelial impairment during systemic inflammation. As expected, Foxf1 was not expressed in lymphatic ECs (Figure 5C), consistent with previously reported lineage‐restricted expression patterns [29]. Collectively, these results demonstrate that Foxf1 and its transcriptional network are markedly suppressed in pulmonary ECs during systemic sepsis, suggesting a mechanistic link between Foxf1 insufficiency and endothelial dysfunction. These observations provide strong rationale for restoring Foxf1 expression in injured pulmonary ECs as a targeted gene‐therapy strategy to enhance vascular repair and mitigate sepsis‐induced lung injury.

### 2.5 Nanoparticle Delivery of FOXF1 mRNA to Pulmonary EC Enhances Lung EC Recovery in ALI/PARDS Mouse Model

**FIGURE 6:**
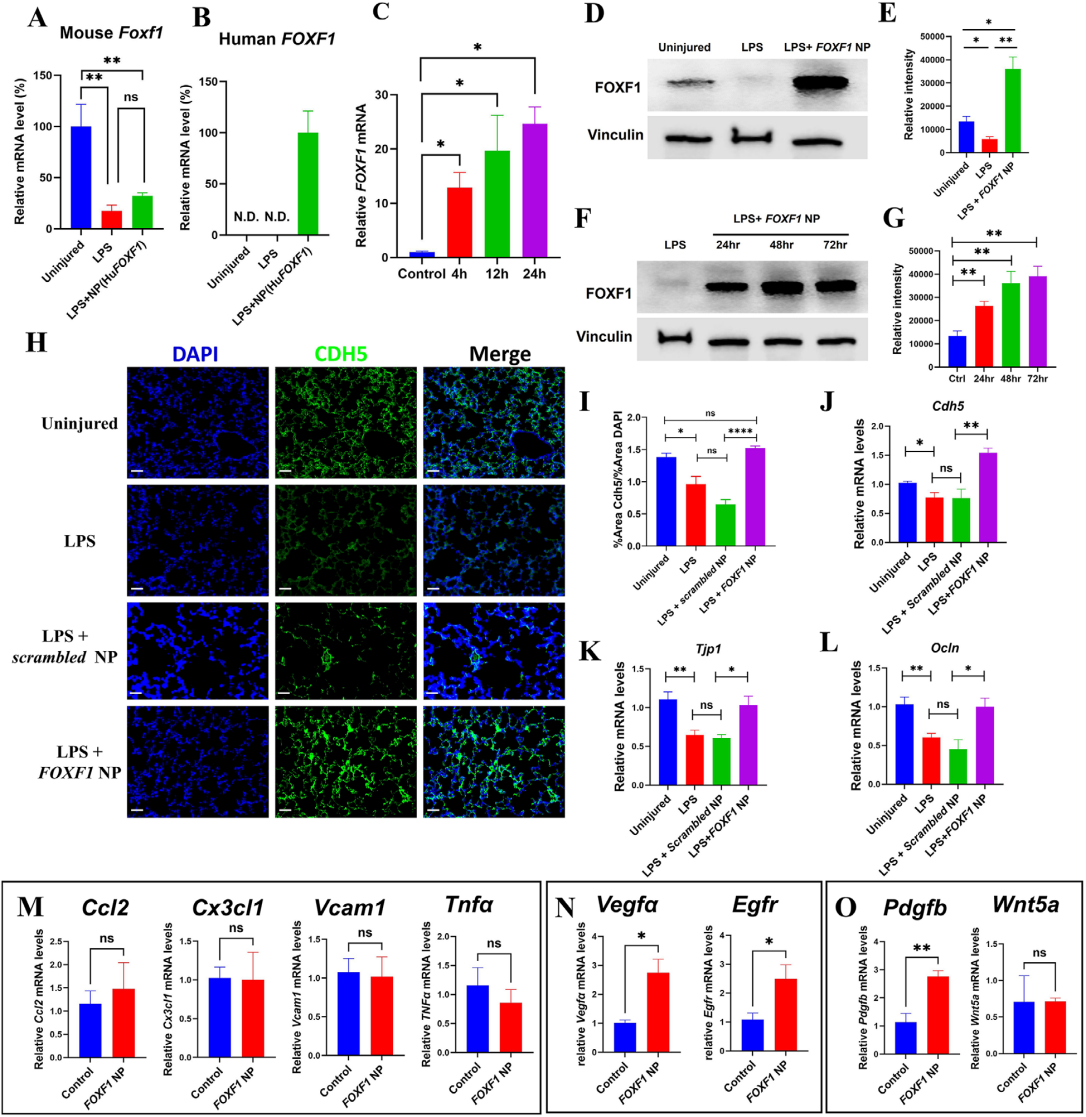
*FOXF1*‐NP delivery enhances pulmonary endothelial recovery in neonatal mice injured with LPS. Mice were injured with LPS at P7 and treated with *FOXF1* NP at P8. (A‐B) Quantification of mouse *Foxf1* and human *FOXF1* mRNAs in lung tissue by qRT‐PCR. Lungs were harvested 4 h after *FOXF1*‐NP delivery. (C) Quantification of *FOXF1* mRNA levels in lung tissue at 4, 8, 12, and 24 h following treatment with *FOXF1* NP. (D‐E) Western blot analysis of FOXF1 protein expression in mouse lung tissues from uninjured, LPS‐treated, and LPS + *FOXF1* NP‐treated groups. (F‐G) Western blot shows FOXF1 protein levels in lung tissue from mice injured with LPS with or without *FOXF1* NP treatment. (H) Representative immunofluorescence images of lung sections stained for the endothelial marker CDH5 (green) and DAPI (blue) in uninjured, LPS‐treated, LPS + *scrambled* NP, and LPS + *FOXF1* NP‐treated groups (Scale bars are 50 µm). (I) Quantification of EC and adherens junction density, shown as the ratio of CDH5‐positive area to DAPI area (J‐L) qRT‐PCR analysis of *Cdh5*, *Tjp1*, *Ocln* mRNAs in lungs of uninjured, LPS‐treated, LPS + *scrambled* NP, and LPS + *FOXF1‐*NP treated mice. (M‐O) mRNAs of genes involved in pulmonary endothelial cell crosstalk: (M) endothelial‐immune crosstalk (*Ccl2*, *Cx3cl1*, *Vcam1*, and *Tnfα*); (N) endothelial‐epithelial crosstalk (*Vegfα* and *Egfr*); and (O) endothelial‐stromal crosstalk (*Pdgfb* and *Wnt5a*). All data are presented as mean ± SEM (n = 5). Statistical significance: *p ≤ 0.05, **p ≤ 0.01, ***p ≤ 0.001, ****p ≤ 0.0001, ns is not significant.

To confirm the *foxf1* mRNA level changes after LPS administration, we proceeded to measure *Foxf1* mRNA levels by qRT‐PCR. As expected, mouse lung *Foxf1* levels were significantly reduced after LPS administration (Figure 6A). Next, we synthesized human *FOXF1* mRNA to distinguish the exogenous and endogenous FOXF1 gene products and encapsulated human *FOXF1* mRNA into nanoparticles for gene therapy. Human *FOXF1* mRNA was detected in lung tissue 4 h after NP delivery through the intravenous route (Figure 6B). Furthermore, the human *FOXF1* mRNA levels continued to increase over the 12‐h and 24‐h time points (Figure 6C). To validate FOXF1 expression at the protein level, we performed Western blots of lung tissue lysates. LPS treatment significantly reduced FOXF1 protein abundance in the lung tissue compared to uninjured controls (Figure 6D‐E). Treatment with FOXF1‐NP increased FOXF1 protein levels in lungs of LPS‐treated mice. Thus, delivering *FOXF1* mRNA with NPs effectively increased FOXF1 protein levels in the lung tissue. In time‐course experiment, FOXF1 protein remained elevated in LPS‐treated lungs during the 3‐day period (Figure 6F‐G). We assessed the therapeutic efficacy of NP delivery of human *FOXF1* mRNA in PARDS because human and mouse *FOXF1* genes are evolutionarily conserved. A day after LPS‐injury (P8), mice received an intravenous injection of NPs containing human *FOXF1* mRNA (*FOXF1* NP) to restore FOXF1 expression in pulmonary ECs. Since LPS administration results in EC loss (Figure 2E), we assessed EC density in *FOXF1* NP treated lungs using immunofluorescence staining for EC marker CDH5, a key transcriptional target of FOXF1 and a critical component of endothelial adherens junctions that maintain vascular integrity [12, 19]. Three days after *FOXF1* NP injection (P12), CDH5 density was significantly higher in the *FOXF1* NP treated group compared with LPS‐injured mice and LPS + *Scrambled* NP group, indicating enhanced endothelial stability (Figure 6H‐I). We also examined CLDN5 (Claudin‐5), the principal protein of tight junctions that regulates paracellular permeability. LPS injury reduced CLDN5 expression in lung tissue, while *FOXF1* NP treatment restored CLDN5 density to near normal (Figure S17). The qRT‐PCR study confirmed that *Cdh5* mRNA was significantly reduced in the LPS‐injured group, whereas *FOXF1* NP treatment effectively restored *Cdh5* mRNA (Figure 6J). In addition to *Cdh5*, some other mRNA critical for EC functions including *Tjp1* and *Ocln* were also explored. Following LPS‐induced injury, both *Tjp1* and *Ocln* expression levels decreased (Figure 6K‐L). However, the *FOXF1* NP treatment group exhibited significantly higher mRNA levels for these genes. Thus, *FOXF1* NP therapy promotes endothelial repair by increasing EC density and enhancing the expression of key EC genes involved in vascular integrity, permeability, and EC stability. We also investigated whether the nanoparticle *FOXF1* mRNA treatment influence the expression of genes critical for EC crosstalk with other pulmonary cell types (Figure 6M–O). Transcripts associated with leukocyte recruitment and inflammatory activation (*Ccl2*, *Cx3cl1*, *Vcam1*, and *Tnfα*) were not affected after *FOXF1* NP treatment (Figure 6M). In contrast, expression of *Vegfα* and *Egfr* was increased in total lung RNA from *FOXF1* NP‐treated mice (Figure 6N). *Pdgfb* is critical for endothelial‐stromal crosstalk by signaling to vascular pericytes to promote their recruitment and stabilization of the pulmonary vasculature [42]. *Pdgfb* mRNA was increased by *FOXF1* NP treatment whereas *Wnt5a* mRNA was unchanged (Figure 6O). These results indicate that *FOXF1* NP treatment influence expression of gene critical for cellular communications after lung injury.

### 2.6 Nanoparticle Delivery of *FOXF1* mRNA to Pulmonary ECs Decreases Endothelial Permeability and Improves Survival of Neonatal Mice After LPS Injury

**FIGURE 7:**
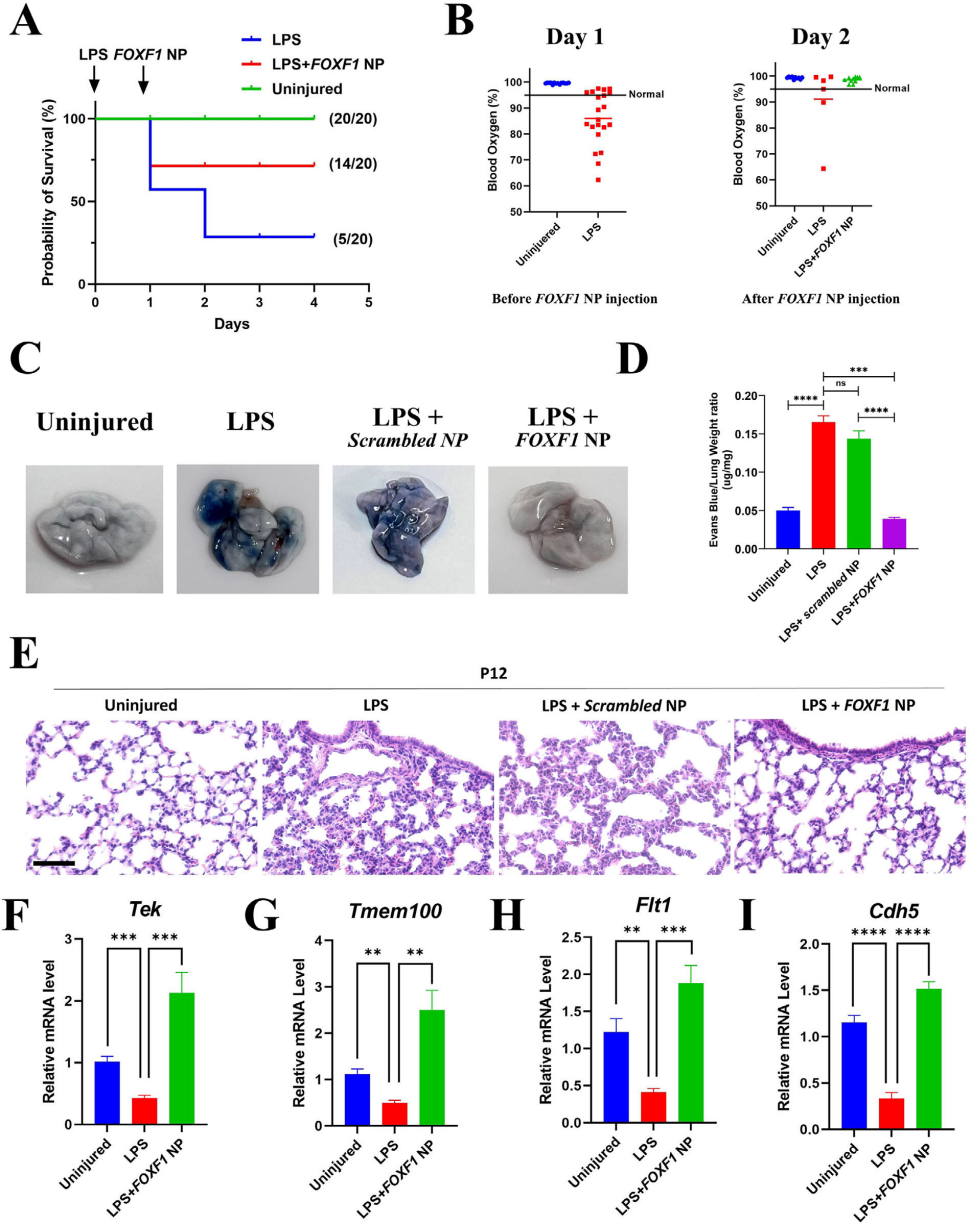
*FOXF1*‐NP therapy improves survival, reduces vascular permeability, and enhances endothelial recovery in neonatal mice with LPS‐induced ALI/PARDS. (A) Kaplan‐Meier survival curves show the survival rates of uninjured, LPS‐injured, and LPS+*FOXF1* NP‐treated neonatal mice. (n = 20 mice per group). (B) Blood oxygen saturation changes 1 day after LPS injury (Day 1). 2‐day time point corresponds to 2 days after LPS injury and 1 day after *FOXF1*‐NP injection. (C) Representative images of lungs from uninjured, LPS‐injured, LPS+*Scrambled* NPs and LPS + *FOXF1* NP‐treated mice 40 mins after injection of Evans Blue *Scrambled* NPs contain no functional nucleic acid. (D) Quantification of Evans blue extravasation in lung tissue 40 mins after injection of Evans Blue. (E) H&E staining was performed using lung sections from uninjured, LPS‐injured, LPS+*Scrambled* NPs and LPS+*FOXF1* NP‐treated mice at P12. Scale bars are 50 µm. (F‐I) qRT‐PCR analysis of *FOXF1* downstream targets (*Tek*, *Flt1*, *Tmem100*, and *Cdh5*) in sorted pulmonary ECs at P9. All data are presented as mean ± SEM (n = 5). Statistical significance: *p ≤ 0.05, **p ≤ 0.01, ***p ≤ 0.001, ****p ≤ 0.0001, ns is not significant.

To evaluate the therapeutic effects of *FOXF1‐*NP in neonatal mice with ALI/PARDS, we monitored survival rates following LPS injection. Compared to uninjured mice, the LPS‐treated group exhibited high mortality, with survival decreasing to 55% on day 1 and 25% on day 2 and day 3 after the injury (Figure 7A). Although delivered 1 day after LPS injury, the *FOXF1*‐NP treatment significantly improved survival, preventing further deaths beyond day 1 and maintaining a 70% survival rate through day 3 (Figure 7A). Blood oxygen saturation (SpO_2_) analysis revealed that 75% of LPS‐treated mice exhibited SpO_2_ levels below 95% (Figure 7B). While some mice recovered by day 2, many succumbed before the second measurement, and 40% of surviving mice continued to exhibit low SpO_2_ levels (Figure 7B). In contrast, *FOXF1‐*NP treatment prevented SpO_2_ decline, and all surviving mice maintained normal SpO_2_ levels by day 2, suggesting a protective effect of *FOXF1*‐NP on oxygenation and respiratory function (Figure 7B). Additionally, *FOXF1*‐NP treatment significantly reduced Evans blue leakage, indicating improved vascular integrity (Figure 7C–D) compared to the LPS‐Injured group without NP treatment. Notably, NP administration without functional nucleic acid failed to improve endothelial integrity, further confirming that *FOXF1* mRNA plays a key role in EC barrier recovery (Figure 7C‐D). Histological analysis of lung tissue further demonstrated that *FOXF1*‐NP therapy mitigated LPS‐induced lung structural damage, leading to improved pulmonary vascular architecture (Figure 7E). To investigate the early molecular response to *FOXF1*‐NP therapy, we sorted pulmonary ECs 16 h after NP delivery and analyzed the expression of *FOXF1* target genes, such as *Tek* (*Tie2*), *Cdh5*, *Tmem100*, and *Flt1* (Figure 7F‐I), all of which play critical roles in neonatal angiogenesis and endothelial barrier function [43, 44, 45, 46]. All tested FOXF1 downstream targets were significantly upregulated, demonstrating a rapid functional response of *FOXF1*‐NPs in injured pulmonary ECs (Figure 7F‐I). To evaluate the long‐term impact of *FOXF1* NP therapy, we conducted a 3‐month study. Histological analysis of H&E‐stained sections showed alveolar simplification in LPS‐injured mice, (Figure S18A‐B). Following *FOXF1* NP therapy, the alveolar simplification was attenuated in LPS‐injured mice. No lung fibrosis was observed in all treatment groups. FlexiVent results showed changes in lung mechanics and respiratory volumes (Figure S18C‐I). Compared to uninjured mice, the LPS group exhibited significant increases in the Maximal lung volume (A), Static compliance (Cst), and Inspiratory capacity (IC), consistent with a hyper‐compliant, over‐distended lung phenotype. After *FOXF1* NP treatment of LPS‐injured mice, the histological and functional parameters were improved with no significant differences compared to uninjured mice (Figure S18). Thus, *FOXF1* NP therapy shows long‐term benefits after neonatal LPS injury. To evaluate the broad therapeutic efficacy of *FOXF1* NP, we tested its effects in an alternative lung injury model which uses bleomycin. Bleomycin exposure is known to cause severe ARDS prior to fibrotic lung remodeling [47, 48, 49]. Bleomycin was delivered intratracheally to induce lung injury, 4 days after injury (the acute lung injury stage), bleomycin‐treated mice exhibited severe lung damage characteristic of ARDS. In contrast, *FOXF1* NP treatment improved lung structure and reduced lung damage (Figure S19A). Flow cytometry analysis demonstrated that bleomycin treatment reduced the percentage of ECs in lung tissue, while the EC percentage was increased after *FOXF1* NP treatment (Figure S19B–C). Additionally, we used qRT‐PCR to measure the mRNA levels of EC markers, including *Ocln*, *Tjp1*, and *Cdh5*. Bleomycin treatment reduced EC mRNAs, whereas *FOXF1* NP treatment increased EC markers (Figure S19D‐F), consistent with data obtained from LPS injury model. These results suggest that *FOXF1* NP treatment has a potential for broad‐spectrum therapeutic efficacy in different ARDS models.

### 2.7 Nanoparticle Delivery of *FOXF1* mRNA Protects Endothelial Cells from Apoptosis and Increases Bcl2 Expression

**FIGURE 8:**
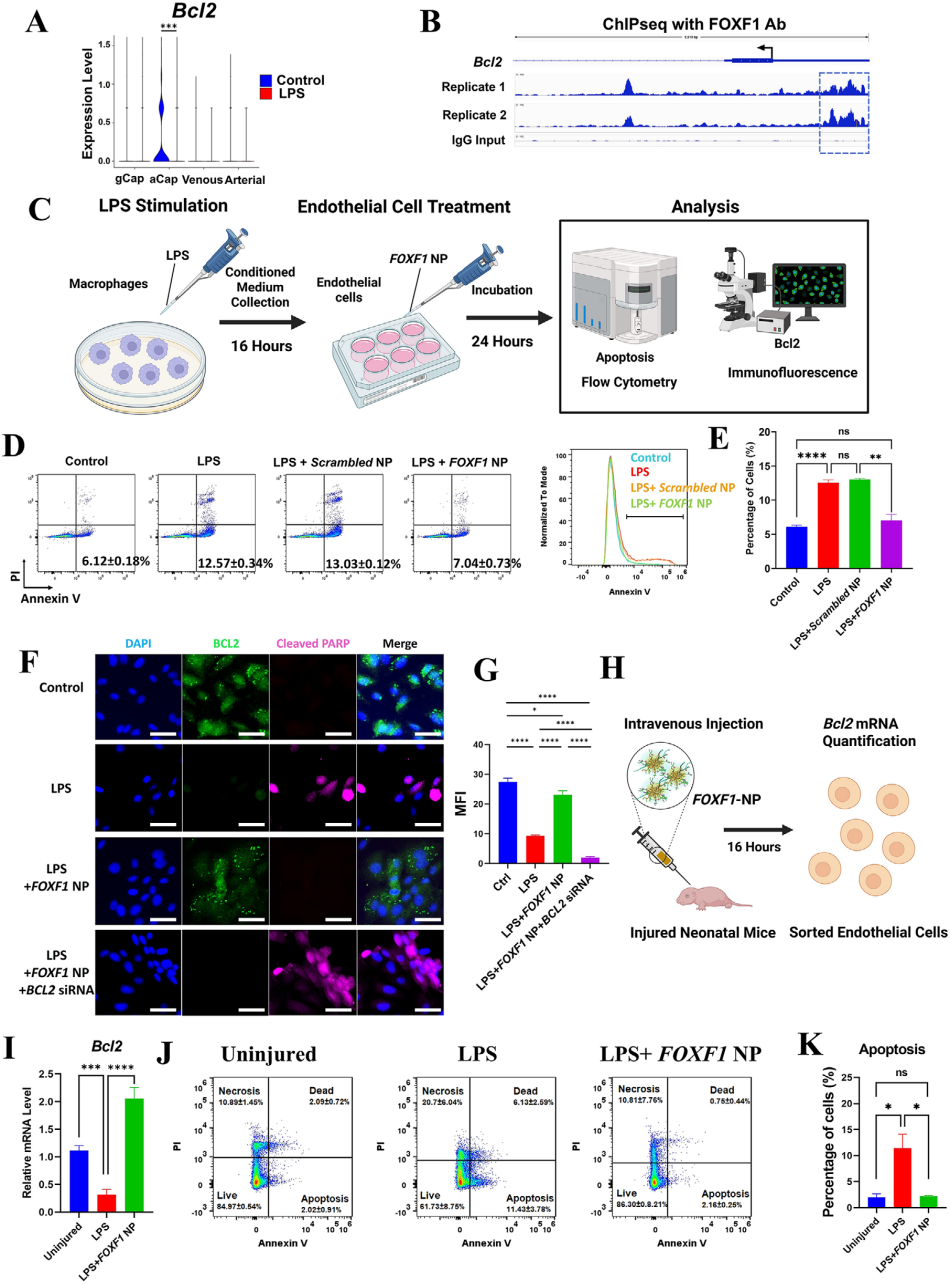
*FOXF1*‐NP treatment reduces pulmonary EC apoptosis. (A) scRNA‑seq shows a significant down‑regulation of the anti‑apoptotic gene *Bcl2* after LPS injury. (B) ChIP‐seq shows FOXF1‐binding peaks in the mouse *Bcl2* promoter. The black arrow represents the direction of the transcription start site. The blue dotted box shows FOXF1‐enriched binding motifs within the *Bcl2* promoter region. Replicates 1 and 2 refer to FOXF1 antibody in 2 independent experiments. IgG input is used as an isotype control. (C) Schematic of the in‐vitro EC apoptosis assay. Inflammatory cytokines secreted by LPS‐stimulated macrophages trigger apoptosis in ECs. Created with BioRender.com. (D) Flow cytometry analysis of EC apoptosis in HUVECs using Annexin V and PI staining. (E) Quantification of EC apoptosis in vitro (n = 5). (F) Representative images of HUVECs treated with culture media secreted by LPS‐stimulated macrophages (LPS), FOXF1 NP, and *BCL2* siRNA. BCL2 is shown with green and Cleaved PARP is shown in magenta, DAPI is blue (Scale bars are 25 µm). (G) The bar graph provides quantification of the BCL2 Mean Fluorescence Intensity (MFI) across treatment groups (n = 30). (H) Schematic of in vivo pulmonary EC apoptosis study using FACS‐sorted pulmonary ECs from injured neonatal mice. Created with BioRender.com. (I) qRT‐PCR analysis of *Bcl2* mRNA in FACS‐sorted pulmonary ECs at P9. (J) Flow cytometry analysis of EC apoptosis at P12 using Annexin V and PI staining. (K) Quantification of EC apoptosis in lung tissue. Data are presented as mean ± SEM (n = 5). Statistical significance: *p ≤ 0.05, **p ≤ 0.01, ***p ≤ 0.001, ****p ≤ 0.0001, ns is not significant.

The immediate improvements in survival rates and SpO_2_ levels following *FOXF1*‐NP administration suggest that the *FOXF1*‐NP treatment not only enhances endothelial recovery but also protects ECs from damage in ALI/PARDS. In the surviving LPS‐treated group, EC percentages began to recover on day 2 after the injury but remained incomplete even by day 4 (Figure S20). In contrast, in the *FOXF1‐*NP treated group, EC percentages remained similar to those in uninjured group (Figure S20), suggesting that the therapeutic benefit may stem from protection against EC apoptosis. Analysis of a publicly available scRNA‑seq dataset revealed a significant down‑regulation of the anti‑apoptotic gene *Bcl2* in alveolar capillary cells (aCap) following LPS exposure (Figure 8A). To investigate the molecular mechanism underlying this protective effect, we analyzed a publicly available FOXF1 ChIP‐Seq dataset [50], and identified *Bcl2*, a well‐characterized anti‐apoptotic gene [51], as a potential direct target of *FOXF1* (Figure 8B). To validate the functional relevance of this pathway, we exposed ECs to inflammatory cytokines secreted by LPS‐stimulated macrophages (Figure 8C). For MFLM‐91U mouse ECs, flow cytometry analysis demonstrated that treatment with inflammatory cytokines significantly increased the proportion of Annexin V‐positive apoptotic ECs. *FOXF1*‐NP transfection into ECs markedly attenuated this effect, reducing the percentage of apoptotic cells (Figure S21A‐C). Immunostaining further confirmed that *FOXF1*‐NP treatment increased BCL2 protein expression in ECs following LPS challenge (Figure S21D). To validate our findings in human ECs, we exposed HUVECs to culture media from LPS‐treated macrophages. Flow cytometry analysis demonstrated that treatment with media containing proinflammatory cytokines significantly increased the proportion of Annexin V‐positive apoptotic cells. However, *FOXF1* NP treatment attenuated this effect, significantly reducing the percentage of apoptotic cells (Figure 8D‐E). To determine if BCL2 is required for the protective effects mediated by *FOXF1* NP, we performed knockdown experiments using *BCL2* siRNA in HUVECs (Figure 8F‐G). Immunostaining showed that LPS decreases BCL2 protein and increased apoptosis in HUVEC cells treated with culture media from activated macrophages. In contrast, NP delivery of *FOXF1* mRNA restored BCL2 expression and reduced apoptosis (Figure 8F‐G). However, when *BCL2* was knocked down with siRNA, the protective effects of *FOXF1* NP were lost, and apoptosis was increased in HUVECs. Thus, BCL2 is required for anti‐apoptotic effect of FOXF1. We next assessed pulmonary endothelial apoptosis in vivo (Figure 8H). Pulmonary ECs were isolated 16 h after *FOXF1*‐NP injection and subjected to qRT‐PCR. As expected, *Bcl2* mRNA levels were markedly reduced after LPS injury (Figure 8I). *FOXF1*‐NP treatment effectively restored Bcl2 expression in FACS‐sorted ECs, confirming that FOXF1 stimulates Bcl2 expression in lung ECs. Furthermore, LPS‐induced apoptosis, as measured by Annexin V staining, was significantly decreased in LPS‐injured ECs after *FOXF1*‐NP treatment (Figure 8J–K). To confirm the expression of BCL2 in the pulmonary vasculature, we performed immunostaining of lung sections (Figure S22). While treatment with LPS significantly reduced BCL2 expression in ECs, *FOXF1* NP treatment increased BCL2 levels in these cells. The reduction in apoptotic ECs correlated with improved EC density in lung tissue, providing strong evidence that *FOXF1*‐NP delivery protects ECs from apoptosis by restoring BCL2 expression.

## 3 Discussion

Pediatric ALI/ARDS remains a major cause of morbidity and mortality, with no effective targeted therapies currently available. Common supportive treatments in the pediatric world usually stem from strategies applied in the adult populations. However, significant differences exist between pediatric and adult populations in terms of lung and chest physiology, as well as cellular senescence. There is an unmet clinical need for novel therapeutic development unique to the pediatric population. Despite significant advances in neonatal care, there is no effective therapeutic strategy targeting the endothelial dysfunction underlying PARDS. Current treatments focus on symptom management rather than the root cause of the disease, leaving a critical gap in clinical care. Recent preclinical investigations have explored various nanoparticle platforms for lung diseases, yet these systems face limitations regarding organ specificity, cellular resolution, and administration routes. Specific cationic polyplexes based on modified PEI have been reported to achieve high efficiency in targeting the microvascular endothelium [31]. However, these NPs lacked total lung specificity, with significant accumulation observed in the kidney and spleen. Another study developed Selective Organ Targeting (SORT) lipid nanoparticles (LNPs). By incorporating a fifth lipid component, these LNPs were engineered to deliver mRNA specifically to lung tissue [52]. While this platform achieves impressive organ‐level specificity, it lacks precise cell‐type resolution within the lung. Quantitative analysis indicated that SORT LNPs accumulate across multiple compartments, including epithelial and immune cells, rather than achieving exclusive delivery to the microvascular endothelium. More importantly, these systems were primarily characterized in healthy, uninjured mouse models and cannot represent lung targeting under disease conditions.

Several nanoparticle platforms have highlighted the potential of inhaled nanoparticles for the treatment of ARDS. Liu et al. designed spike‐conjugated gold nanoparticles that target epithelial cells through ACE2 and L‐SIGN receptors. This design inhibited the p38α MAPK pathway, reducing proinflammatory cytokines in BALF and restoring lung function 6 h after inhalation [53]. Sun et al. developed a lipid‐based nanoparticle (Pac‐lipo) to deliver the natural flavonoid pachypodol. These particles diffuse highly efficiently through respiratory mucins, effectively repairing epithelial barriers after lung injury and suppressing the TLR4‐MyD88‐NF‐κB/MAPK signaling pathway [54]. Despite these advancements, both NP platforms are restricted by their administration routes. These studies used direct lung injury models induced via local administration of LPS or HCl, rather than systemic sepsis models, in which pulmonary ECs are damaged. Inhalation or intratracheal deliveries primarily target the airway and alveolar epithelial compartments and are unable to directly target the pulmonary capillary endothelium. Since these studies used adult animal models, they do not reflect the developmental signaling and unique vulnerabilities of neonatal and pediatric populations. Our nanoparticle mRNA delivery system addresses these limitations by demonstrating high‐efficiency targeting of the lung endothelium in neonatal mice. As a polymeric platform, it offers lower production costs and a more straightforward scale‐up process than complex liposomal systems, while the use of biodegradable polymers ensures a favorable safety profile compared to conventional high‐molecular‐weight carriers. By enabling the precise delivery of therapeutic agents to lung endothelial cells, our NP system provides a roadmap for the transition to future precision therapies in pediatric ARDS.

Since the pulmonary endothelium plays a crucial role in the pathogenesis and resolution of ALI/PARDS, we were able to show that therapies targeting the pulmonary EC are effective in rescuing from the PARDS phenotype. Consistent with our hypothesis, we revealed that FOXF1 expression is significantly reduced in our PARDS model, which was associated with EC dysfunction and apoptosis. EC‐targeted exogenous FOXF1 mRNA administration ameliorates the phenotype caused by ALI/PARDS. We chose to deliver *FOXF1* mRNA due to the transcription factor's role in angiogenesis, maintenance of the EC barrier integrity, and promotion of lung repair after injury [19,55]. Our results show that FOXF1 levels are significantly decreased following acute lung injury, suggesting that its restoration via *FOXF1* mRNA delivery holds high therapeutic potential. While *FOXF1* is expressed in both adult and neonatal pulmonary endothelial cells, *FOXF1* NP treatment offers a unique advantage in neonates because FOXF1 is required for angiogenesis and vascular development in the developing lung [56]. Since neonatal PARDS often leads to permanent alveolar simplification and impaired lung development, our findings suggest that *FOXF1* NP treatment can improve long‐term functional outcomes on patients with PARDS. In addition, downstream targets of FOXF1, such as Tek (Tie2), Flt1 (VEGFR1), Cdh5 (VE cadherin), and Tmem100, were assessed due to their importance in EC function [45,57, 58, 59]. Expression of these genes was found significantly reduced in our ALI/PARDS model and associated with the loss of FOXF1 in EC subtypes. EC‐targeted delivery of *FOXF1* mRNA restored expression of *FOXF1* and its target genes.

Naked mRNA molecules are susceptible to biodegradation, requiring a delivery vehicle to prolong bioavailability. Currently available delivery vehicles are, mostly, nonspecific with broad biodistribution and unknown toxicity profiles. An important contribution of our manuscript is that we achieved a precise delivery of *FOXF1* mRNA into lung ECs using a PBAE‐based NP vehicle. This was accomplished by precisely controlling the size, avoiding NP accumulation in the spleen and liver, and by controlling the charge of the PBAE polymer. In our previous study, we screened a wide range of PBAE formulations and identified a specific PBAE‐NP structure capable of targeting pulmonary ECs [34]. This targeting is achieved through passive mechanisms driven by specific physicochemical properties. A key feature of this formulation is its unique hydrophobic core, which optimizes targeting efficiency by influencing NP stability, serum protein interactions, and cell membrane affinity [34]. In this published study, when the hydrophobic monomer types or ratios were altered, the NPs lost their targeting specificity to the lung tissue. NP size is a critical factor in pulmonary EC targeting. Published data showed that NPs with diameters exceeding 130 nm are primarily sequestered by the spleen, while reducing the size to 100 nm significantly increased lung targeting efficiency. This is because larger NPs are removed by the mononuclear phagocyte system (MPS), while smaller particles are filtered by the renal system [60]. Additionally, the surface charge of the NPs plays an important role in interacting with pulmonary ECs [61]. Since the EC surface is coated with a negatively charged glycocalyx, which consists of an anionic network of glycosaminoglycans, glycolipids, and glycoproteins, positively charged NPs interact more readily with this surface to enhance cellular uptake [62]. Because the lungs possess a dense network of capillary ECs, this electrostatic interaction enhances EC uptake and reduces the chance of NPs targeting other pulmonary cell types. The net effect of these physicochemical properties allows the NPs to selectively target the pulmonary ECs in PARDS. This is the first NP delivery system capable of delivering mRNAs precisely into pulmonary ECs without affecting endothelial cells of other organs in the body.

Our NPs are biodegradable due to the presence of ester groups, contributing to their high biocompatibility and low toxicity. Complete metabolic profile testing did not reveal any indication of kidney and liver function abnormalities, and hematologic assessment showed normal ranges for blood cells. Our results reaffirm the non‐toxic nature of the NPs and therefore, the NPs can be used to deliver any mRNA or DNA plasmids into pulmonary ECs for gene therapies in lung diseases. The biodegradable property of PBAE NPs also ensures rapid cargo release as evidenced by increased human *FOXF1* mRNA levels in the lung tissue. Lung‐specific expression of luciferase and human *FOXF1* mRNAs are consistent with rapid and efficient in vivo transfection capability of the NP system.

The immediate improvements in EC numbers and function suggest that FOXF1 not only enhances endothelial recovery after injury but also protects ECs from apoptosis. The anti‐apoptotic factor Bcl2 was identified as a downstream target of *FOXF1* and was significantly reduced in alveolar capillary ECs of LPS‐injured mice. *FOXF1*‐NP administration restored Bcl2 expression, protecting ECs from cell death in ALI/PARDS. Our results demonstrate that *FOXF1*‐NP therapy after LPS injury decreases endothelial apoptosis, enhances endothelial barrier function, and increases arterial oxygenation highlighting its potential as a promising treatment for human ALI/PARDS. One of the limitations of our study is the reliance on mouse models of PARDS. Interspecies differences in pulmonary endothelial biology, immune responses, and nanoparticle biodistribution may affect the direct translation of our findings to humans. Future studies are needed to evaluate the *FOXF1* NP system in humanized mouse models and non‐human primates. These models of PARDS will provide a more accurate evaluation of the therapeutic potential and help bridge the gap between our current findings in mice and future clinical applications.

The transition of pulmonary nanomedicines from laboratory settings to clinical application is limited by various technical and regulatory obstacles. A primary barrier is the difficulty of achieving large‐scale production for nanoparticles with complex architectures. These complex designs are highly sensitive to production parameters. For example, industrial scale‐up usually requires higher throughput and increased flow rates, which can alter hydrodynamic variables and lead to nonuniform self‐assembly, aggregation, and inconsistent therapeutic loading. Furthermore, the use of high‐cost, low‐yield biological reagents can significantly increase preparation costs and reduce production efficiency. In addition to industrial manufacturing, regulatory requirements are another limitation. Because NP structural complexity prevents the application of universal standardized tests, the U.S. Food and Drug Administration (FDA) and the European Medicines Agency (EMA) utilize case‐by‐case methodologies to evaluate nanomedicines [63]. To address these barriers, keeping nanoparticle designs simple and manufacturing processes straightforward is essential for industrial feasibility and the regulatory process. One fundamental approach in manufacturing is the use of Quality by Design (QbD) principles during early process development. This framework identifies high‐risk parameters to establish a stable manufacturing process and uses Critical Quality Attributes (CQAs) as measurable benchmarks for clinical performance [64].

## 4 Conclusion

In summary, we used a mouse model of ALI/ARDS to enhance endothelial barrier integrity, decrease EC apoptosis and improve mice survival. We were able to achieve this by selective targeting of the pulmonary EC via a lung‐specific NP delivery system capable of delivering *FOXF1* mRNA. More importantly, the *FOXF1*‐NP treatment exhibits rapid therapeutic benefits with no evident toxicity. Given excellent lung specificity, efficacy, and safety of this treatment, *FOXF1*‐NPs hold great promise for future clinical applications in the treatment of ALI/PARDS.

## 5 Experimental Section

### 5.1 Reagents, Chemicals, and Nucleic Acids

Lipopolysaccharide from Escherichia coli O111:B4 (L2630) was purchased from Sigma‐Aldrich (St. Louis, MO, USA). All materials for PBAE polymer and NP synthesis were purchased from Sigma‐Aldrich (St. Louis, MO, USA), including bisphenol A ethoxylate (EO/phenol) diacrylate, 4,4′‐trimethylenedipiperidine, 6‐amino‐1‐hexanol, polyethylenimine (PEI), methoxypolyethylene glycol amine (mPEG), and pentadecafluorooctanoyl chloride. D‐Luciferin (Potassium Salt) was purchased from Gold Biotechnology (St. Louis, MO, USA). Cyanine5 NHS ester (Cy5) was purchased from Lumiprobe (Hunt Valley, MD, USA). GFP, luciferase, and *FOXF1* mRNAs were purchased at RNACore of Houston Methodist Research Institute (Houston, TX, USA). The sequence of the *FOXF1* mRNA is *AUGUCUUCGGCGCCCGAGAAGCAGCAGCCACCGCACGGCGGCGGCGGCGGCGGCGGCGGGGGAGGCGGCGCGGCCAUGGACCCCGCGUCGUCCGGCCCGUCCAAGGCCAAGAAGACCAACGCCGGCAUCCGGCGCCCGGAGAAGCCGCCCUAUUCCUACAUCGCGCUCAUCGUCAUGGCCAUCCAGAGUUCACCCACCAAGCGCCUGACGCUGAGCGAGAUCUACCAGUUCCUGCAGAGCCGCUUCCCCUUCUUCCGGGGCUCCUACCAGGGCUGGAAGAACUCCGUGCGCCACAACCUCUCGCUCAACGAGUGCUUCAUCAAGCUACCCAAGGGCCUUGGGCGGCCCGGCAAGGGCCACUACUGGACCAUCGACCCGGCCAGCGAGUUCAUGUUCGAGGAGGGCUCCUUUCGGCGGCGGCCGCGCGGCUUCCGAAGGAAAUGCCAGGCGCUCAAGCCCAUGUACAGCAUGAUGAACGGGCUCGGCUUCAACCACCUCCCGGACACCUACGGCUUCCAGGGCUCGGCCGGCGGCCUCUCGUGCCCGCCCAACAGCCUGGCGCUGGAGGGCGGCCUGGGCAUGAUGAACGGCCACUUGCCGGGCAACGUGGACGGCAUGGCCCUGCCCAGCCACUCGGUGCCCCACCUGCCUUCCAACGGCGGCCACUCGUACAUGGGCGGCUGCGGCGGCGCGGCGGCCGGCGAGUACCCGCACCACGACAGCUCGGUGCCCGCCUCCCCGCUGCUGCCCACCGGCGCCGGUGGGGUCAUGGAGCCGCACGCCGUCUACUCGGGCUCGGCGGCGGCCUGGCCGCCCUCGGCGUCCGCGGCGCUCAACAGCGGCGCCUCUUAUAUCAAGCAGCAGCCCCUGUCCCCCUGUAACCCCGCGGCCAACCCCCUGUCCGGCAGCCUCUCCACGCACUCCCUGGAGCAGCCGUAUCUGCACCAGAACAGCCACAACGCCCCAGCCGAGCUGCAAGGCAUCCCGCGGUAUCACUCGCAGUCGCCCAGCAUGUGUGACCGAAAGGAGUUUGUCUUCUCUUUCAACGCCAUGGCGUCCUCUUCCAUGCACUCGGCCGGCGGGGGCUCCUACUACCACCAGCAGGUCACCUACCAAGACAUCAAGCCUUGCGUGAUGUAG*. The pALD‐Nanoplasmid‐CAG‐eGFP was purchased from Aldevron (Fargo, ND, USA). Mouse Complement C5 ELISA kit was purchased from Abcam (catalog #ab264609). All antibodies and kits used for flow cytometry and immunostaining were purchased from BioLegend (San Diego, CA, USA), BD Bioscience (Franklin Lakes, NJ, USA), MilliporeSigma (Burlington, MA, USA), Santa Cruz (Dallas, TX, USA) and ThermoFisher (eBioscience) and listed in table S1. All antibodies used in this study have been validated and published for neonatal lungs and secondary‐only controls were used to confirm the absence of non‐specific secondary antibody binding in the IF images.

### 5.2 Mice and Sepsis Model

All animal experiments were approved by the University of Arizona Institutional Animal Care and Use Committee (IACUC) and conducted following NIH guidelines for laboratory animal care and safety. Wild‐type CD1 mice (Charles River Laboratories) were used. ALI/PARDS was induced in postnatal day 7 (P7) mice via intraperitoneal (I.P.) injection of LPS (5 mg/kg) (Figure 1A). Sixteen h later (P8), NPs containing either Luciferase mRNA or FOXF1 mRNA were administered intravenously via the retro‐orbital sinus. Tissues were analyzed between P9‐P12 to assess recovery after injury (Figure 1B). Clinical toxicity of NPs was analyzed using IDEXX BioAnalytics (North Grafton, MA, USA). Peripheral blood was collected as described previously [28, 29, 34]. Evans blue dye (20 mg/kg) was injected retro‐orbitally 40 min before tissue collection. The vasculature was perfused to remove intravascular dye. Evans blue concentration in the lung tissue was determined by absorbance at 620 and 740 nm, normalized to a standard curve, and expressed as the Evans blue‐to‐lung weight ratio to assess endothelial permeability. Bronchoalveolar lavage fluid (BALF) was collected via tracheal instillation and retrieval of cold PBS 30 min after LPS injection. Blood oxygen saturation (SpO_2_) was measured using a MouseOX Plus pulse oximeter (STARR Life Sciences, Oakmont, PA, USA), with data analyzed using MouseOX Plus 1.6.x software. Tissue and BALF cellular analyses were performed using flow cytometry. The Systemic Inflammation Response Index (SIRI) is calculated using the following equation:

$$SIRI=\frac{\mathrm{Neutrophils}\;\times \;\mathrm{Monocytes}}{\mathrm{Lymphocytes}}$$

### 5.3 Nanoparticle Synthesis and mRNA Encapsulation

PBAE polymers were synthesized via Michael Addition (Figure S1) [34]. Bisphenol A ethoxylate diacrylate and 6‐amino‐1‐hexanol were mixed in anhydrous DMSO at 90°C for 24 h, followed by 4,4′‐trimethylenedipiperidine addition at 50°C for 24 h. Excess end‐capping amines, including fluorinated PEI and mPEG, were added and reacted at 40°C for another 24 h. Polymers were dialyzed against Milli‐Q water using 3.5 kDa tubing (Thermo Scientific) to remove excess reactants and DMSO. For mRNA encapsulation, PBAE polymers were vortex‐mixed with mRNA. Fluorescent NPs were prepared by mixing NHS‐functionalized fluorophores with PBAE polymers at room temperature. NP hydrodynamic diameter and surface potential were measured using dynamic light scattering (DLS) (Zetasizer Ultra, Malvern, UK). Gel electrophoresis was performed using 0.8% TBE‐based agarose gels and imaged with an Azure Imaging System 200.

### 5.4 In Vitro Study

Mouse fetal lung endothelial cells (MFLM‐91U) were purchased from Seven Hills Bioreagents (Cincinnati, OH, USA). Human Umbilical Vein Endothelial Cells (HUVEC) were purchased from Lonza (Catalog #: C2517A). MFLM‐91U cells were cultured in Dulbecco's Modified Eagle Medium (DMEM, Gibco) supplemented with 10% fetal bovine serum (FBS, Gibco) and 1% v/v penicillin streptomycin (Invitrogen) in a controlled environment at 37°C and 5% CO_2_. HUVECs were cultured in EGM‐2 Endothelial Cell Growth Medium‐2 with supplements in the kit (Lonza, Catalog #: CC‐3162). The cells were seeded into 24‐well tissue culture plates 24 h before transfection. GFP mRNA, GFP plasmid and PBAE polymers were diluted in 100 ng/µl and 1 µg/µl, separately, followed by mixing in the OPTI‐MEM (Gibco) at different ratios. NPs encapsulated with GFP mRNA or GFP plasmid were added to the cells at a concentration of 500 ng/well. Medium was replaced with fresh DMEM medium with 10% FBS 4 h after transfection. The efficiency of transfection was determined at day 1–3 after transfection. Fluorescence images were taken at the highest transfection time point (1 day after mRNA transfection, 2 days after plasmid transfection) using Invitrogen EVOS FL Auto 2 microscope. For the in‑vitro apoptosis assay, RAW 264.7 macrophages were stimulated with LPS (100 ng/mL) for 16 h to induce cytokine production. The conditioned medium from treated or untreated macrophages was collected and added to ECs. The ECs were then co‑incubated with nanoparticles containing *FOXF1* mRNA or a nonfunctional empty plasmid. Flow cytometry and immunofluorescence analyses were performed to assess apoptosis in the ECs and to evaluate changes in *BCL*‑2 expression. The ON‐TARGETplus 2.0 *BCL2* siRNA (purchased from Horizon) was used for *BCL2* knockdown following the user guide. The Cleaved PARP Rabbit Monoclonal Antibody (purchased from Cell Signaling, #5625) was used to stain apoptotic cells.

### 5.5 In Vivo and Ex Vivo Imaging

The Revvity IVIS lumina LT series III (Waltham, MA, USA) was used to capture the In vivo and Ex vivo images in NPs biodistribution studies. P8 mice with or without injury were injected intraperitoneally with 50 µL of D‐luciferin (30 mg/mL) 4 h after i.v. delivery of NPs containing Luciferase mRNA. For ex vivo study, the heart, liver, spleen, lung, and kidney were collected for luminescence imaging. The luminescence intensity was quantified using the Living Image software.

### 5.6 Flow Cytometry and Flow Sorting

Flow cytometry was performed on single‐cell suspensions from enzyme‐digested lung tissue as described [65]. Live cells were identified using 7‐AAD, except in apoptosis studies, where FITC‐Annexin V/PI staining was used. Antibodies are listed in Table S1. FACS analysis was performed using Attune NxT Flow Cytometer. EC sorting was done using a SONY SH800S Cell Sorter, collecting ∼90,000 cells for further analysis. Data were analyzed with FlowJo software.

### 5.7 Quantitative RT‐PCR

RNA was extracted from tissue, BALF, and sorted CD31+CD45 endothelial cells from uninjured, LPS‐treated, and LPS+FOXF1 NP‐treated mice using Quick‐RNA Microprep or Miniprep Kits (Zymo Research, CA). RNA was reverse transcribed using the iScript cDNA Synthesis Kit (Bio‐Rad). qRT‐PCR was performed on a QuantStudio 3 system using SYBR Green or TaqMan assays (Table S2). Each sample was run in triplicate to minimize variability. mRNA levels were normalized to β‐actin mRNA or GAPDH mRNA.

### 5.8 Histology and Immunostaining

Lung sections were stained with hematoxylin and eosin (H&E) according to manufacturer's protocol (ScyTek Laboratories, UT, USA). Immunostaining was performed using antibodies shown in the table S1. Brightfield images and fluorescence images were captured using Invitrogen EVOS FL Auto 2 microscope. Z‐stack fluorescence images for 3D reconstruction were obtained using ZEISS Apotome 3 imaging system. The quantification of fluorescence area and intensity was calculated using the FIJI (ImageJ) software.

### 5.9 scRNAseq and ChipSeq

The data were accessed from the GSE148499 dataset. Basal (Control) and Day2_post LPS (LPS) sets were utilized for analysis [66]. Briefly, separate Seurat objects were created, normalized, scaled and variable features were found using SCT transform with default parameters and doublet removal as described [67]. To perform unsupervised clustering analysis, FindNeighbors (dims 1:20), FindClusters (resolution = 0.1), and RunUMAP (dims 1:20) were used. Cell clusters were visualized using dotplots. Aberrant non‐EC clusters with no Pecam1 expression were removed before integration. The objects were merged with harmony and processed as described [68]. To identify clusters, we used previously published datasets [69] and Lungmap (lungmap.net) [70]. Expression of *Tek*, *Flt1*, *Tmem100* and *Bcl2* was visualized using VlnPlot_scCustom as described [17]. Statistical significance for each mRNA was calculated using the pairwise Wilcox test with a correction using the Benjamini‐Hochberg method. For ChIP‐seq analysis of *Bcl2*, we used the publicly available dataset GSE77951. FOXF1‐binding picks were visualized using IGV_2.18.4 as described [50].

### 5.10 Statistical Analysis

Statistical significance was determined using Student's t‐test (Prism software), with P < 0.05 considered significant. For animal number less than 6, nonparametric Mann‐Whitney u test was used. Data were presented as mean ± SEM. Kaplan‐Meier analysis was used to assess survival.

## Author Contributions

**Zicheng Deng**: Conceptualization, Methodology, Validation, Formal analysis, Investigation, Data curation, Visualization, Writing – original draft. **Wen Gao**: Methodology, Investigation, Data curation, Visualization. **Jonathan Do**: Methodology, Investigation, Data curation. **Danli Lu**: Investigation, Data curation. **Ying‐Wei Lan**: Methodology, Investigation, Data curation. **Andreas Damianos**: Writing – Review & Editing. **Gautam Verma**: Investigation. **Ali Al Siraj**: Resources. **Isabella Lowry**: Resources. **Lance Peter**: Resources. **Nicholas E. Banovich**: Resources. **Fei Sun**: Data curation. **Hua He**: Data curation. **Tanya V. Kalin**: Resources, Supervision. **Vladimir V. Kalinichenko**: Conceptualization, Validation, Resources, Project administration, Writing – Review & Editing, Supervision, Funding acquisition.

## Funding Sources

This study is supported by the National Heart, Lung, and Blood Institute (NHLBI) at the National Institutes of Health (NIH). Grant numbers: HL141174, HL149631, HL152973, HL188210.

## Conflicts of Interest

The authors declare no conflicts of interest.

## Supporting information




**Supporting File**: advs77813‐sup‐0001‐SuppMat.docx.

## Data Availability

The data that support the findings of this study are available from the corresponding author upon reasonable request.
