# Supplementary material for: Developing Highly Effective Nanoparticle mRNA Therapeutic for Pediatric Acute Respiratory Distress Syndrome
Source: Adv Sci (Weinh). 2026 Sep 17:e77813. Online ahead of print. doi: 10.1002/advs.77813 (PMC13584808; doi:10.1002/advs.77813)
Supplement: Supplementary file 1 [file ADVS-9999-e77813-s001.docx]

Supporting Information

**Developing Highly Effective Nanoparticle mRNA Therapeutic for Pediatric Acute Respiratory Distress Syndrome**

*Zicheng Deng ^1^; Wen Gao ^1^; Jonathan Do ^1^;* *Danli Lu ^1^; Ying-Wei Lan ^1^; Andreas Damianos ^1,2^,* *Gautam Verma ^1^; Ali Al Siraj ^1^, Isabella Lowry ^1^, Lance Peter ^3^, Nicholas E. Banovich ^3^, Fei Sun ^4,5,6^, Hua He ^4,5,6^, Tanya V. Kalin ^1,4^, Vladimir V. Kalinichenko ^1, 2^ **

^1^ Phoenix Children’s Research Institute, Department of Child Health, University of Arizona College of Medicine – Phoenix

^2^ Division of Neonatology, Phoenix Children’s Hospital

^3^ Division of Bioinnovation and Genome Sciences, Translational Genomics Research Institute

^4^ Key Laboratory of Birth Defects and Related Diseases of Women and Children of MOE, West China Second University Hospital, Sichuan University, Chengdu, Sichuan, China

^5^ NHC Key Laboratory of Chronobiology, Sichuan University, Chengdu, Sichuan, China

^6^ The Joint Laboratory for Lung Development and Related Diseases of West China Second University Hospital, Sichuan University and School of Life Sciences of Fudan University, Chengdu, Sichuan, China

^7^ Phoenix Children’s Center for Cancer and Blood Disorders

*Correspondence: Vladimir V. Kalinichenko

**Figure S1**. Synthesis of the PBAE backbones via Michael addition reactions and the end-caps/fluorescent dye use in PBAE polymer synthesis.

**Figure S2**. qRT-PCR analysis shows mRNA expression of pro-inflammatory cytokines (*Il1, Il6, Tnfα*) in BALF cells 30 minutes after LPS injection. Data are normalized to *β-actin* mRNA and presented as mean ± SEM (n = 5). Statistical significance: **p ≤ 0.01, ***p ≤ 0.001.

**Figure S3**. (A) Body weight change (%) in LPS injured neonatal mice 1 day after LPS injection compared to uninjured controls. (B) Total immune cell count in BALF cytospins from uninjured and LPS-injured mice. (C) Representative images of BALF cytospins stained with Diff-Quik at 20× and 40× magnification show increased cell numbers in the BALF from LPS-treated mice. Data are presented as mean ± SEM (n = 5). Statistical significance: **p ≤ 0.01, ***p ≤ 0.001.

**Figure S4**. Gating strategy for the identification of neutrophils (CD45+Ly6G+Ly6C+) and macrophages (CD45+Ly6C-F4/80+) in BALF. Eosinophils were excluded by gating out (SiglecF+CD11b+) cells.

**Figure S5**. (A) Flow cytometry analysis of hematopoietic cell populations (total cells, macrophages, and neutrophils) in BALF from uninjured and LPS-treated mice. (B) Quantification of cell percentages in BALF. Data are presented as mean ± SEM (n = 5). Statistical significance: **p ≤ 0.01, ****p ≤ 0.0001, ns is not significant.

**Figure S6**. qRT-PCR analysis shows mRNA expression of pro-inflammatory cytokines (*Il1, Il6, Tnfα*) in lung tissue 30 minutes after LPS injection. Data are normalized to *β-actin* mRNA and presented as mean ± SEM (n = 5). Statistical significance: ***p ≤ 0.001, ****p ≤ 0.0001.

**Figure S7**. Gating strategy for the identification of cell lineage populations (CD31+ CD45- as endothelial cells, CD45+ CD31- as hematopoietic cells, CD326+ CD31- CD45- as epithelial cells, CD140a+ CD31- CD45- as fibroblasts, NG2+ CD140a- CD31- CD45- as pericytes, and NG2- CD140a- CD31− CD45− CD326-as lineage-negative cells) using whole-lung single cell suspensions.

**Figure S8**. qRT-PCR analysis of endothelial markers *Tjp1, Ocln, Cldn5*. Total mRNA was prepared from lungs of uninjured and LPS-injured mice 1 day after LPS i.p. administration. All data are normalized to *GAPDH* mRNA and presented as mean ± SEM (n = 5). Statistical significance: *p ≤ 0.05, **p ≤ 0.01, ****p ≤ 0.0001.

**Figure S9**. Gating strategy for the identification of cell lineage populations (CD31+ CD45- as endothelial cell and CD45+ CD31- as hematopoietic cell) and their changes before and after LPS treatment. Cells were prepared from liver (A) and kidney (B). Data are presented as mean ± SEM (n = 3). Statistical significance: *p ≤ 0.05, ns is not significant.

**Figure S10.** Polydispersity index (PDI) of NP before and after assembly over time. The PDI was characterized for NPs synthesized in 6 different batches and stored for different periods of time. Data are presented as mean ± SD.

**Figure S11.** H&E staining of major organs from control and NP‑treated groups shows no significant morphological alterations (Scale bar = 50 μm).

**Figure S12.** Evaluation of systemic immune response following repeated NP administrations. (A) Standard curve for determining complement C5 concentration. (B) Concentration of complement C5 (ng/ml) in the blood of untreated and NP-treated groups. (C) Immune cell counts, including white blood cell (WBC), neutrophils, lymphocytes, monocytes, eosinophils, and basophils, in the NP-treated and untreated groups. (D) Systemic inflammation response index (SIRI) values comparing NP-treated and untreated groups. Data are presented as mean ± SEM (n=5); ns indicates no significant difference.

**Figure S13.** In vitro immunofluorescence images tracking Cy5‑labeled NPs demonstrate their localization within 91U lung ECs (scale bar = 25 μm).

**Figure S14**. Representative immunofluorescence images of lung sections stained for CD31 (red), αSMA (green), and DAPI (blue). NPs (white) are detected in endothelial cells of small pulmonary arteries and veins located in alveolar regions (Scale bar=50 μm).

**Figure S15**. Dose-response and biodistribution of the NPs in neonatal mice. (A) Representative bioluminescent images of murine organs (lung, spleen, liver, heart, and kidney) in neonatal CD-1 mice following administration of varying doses of Luciferase mRNA-loaded NPs (0.1-0.5 μg/g of body weight). (B) Quantification of average radiance across different organs. Data are presented as mean ± SD (n = 3).

**Figure S16.** IVIS images compare intravenous (I.V.) and intratracheal (I.T.) nanoparticle administration. (A) Representative luminescence image; I.V. injection shows significantly higher luciferase activity than I.T. injection. (B) Quantitation of average luminescence intensity in lung tissue for I.V. and I.T. administrations. Data are presented as mean ± SEM (n=3). Statistical significance: **p ≤ 0.01.

**Figure S17.** (A) Representative immunofluorescence images of lung sections stained for the endothelial marker CLDN5 (green) and DAPI (blue) in uninjured, LPS-treated, and LPS + *FOXF1* NP-treated groups (Scale bars are 50 µm). (B) Quantification of EC and tight junction density, shown as the ratio of CLDN5-positive area to DAPI area.

**Figure S18. Long-term effects of *FOXF1* NP treatment 3 months after LPS injury.** (A) Representative H&E staining of lung tissue from uninjured mice, mice treated with LPS, and mice treated with LPS + *FOXF1* NP. (B) Mean Linear Intercept (MLI) calculated from H&E-stained images (n=5). (C-E) Lung mechanical and functional parameters have been determined using flexiVent analysis: Maximal lung volume (A), Static compliance (Cst), Inspiratory capacity (IC). Data are presented as mean ± SEM (n=4-6). Statistical significance: *p≤0.05, **p ≤ 0.01. ns is not significant.

**Figure S19. *FOXF1* NP therapy increases the number of ECs after bleomycin-induced lung injury.** (A) Representative H&E staining of lung tissues from uninjured, bleomycin (Bleo)-treated, and Bleo + *FOXF1* NP treated groups. Arrowheads indicate areas of inflammatory cell infiltration and structural damage in the Bleo group. Scale Bar = 50 μm. (B) Representative flow cytometry plots show endothelial (CD31+ CD45-) and hematopoietic (CD31- CD45+) cell populations in uninjured, bleomycin (Bleo)-treated, and Bleo + *FOXF1* NP treated mouse lungs. (C) Quantification of endothelial populations based on flow cytometry measurements. (D-F) qRT-PCR shows mRNAs *Ocln*, *Tjp1*, and *Cdh5* in lung tissues. Data normalized to β-actin or GAPDH and are presented as mean ± SEM (n = 5). Statistical significance: *p ≤ 0.05, **p ≤ 0.01, ***p ≤ 0.001.

**Figure S20.** Bar graph shows changes in percentages of pulmonary endothelial cells in uninjured, LPS-injured and LPS+*FOXF1* NP groups on different days. Flow cytometry was used to identify the percentages of endothelial cells among total live cells. All data are presented as mean ± SEM (n = 5). Statistical significance: *p ≤ 0.05.

**Figure S21.** (A-B) Flow cytometry analysis of EC apoptosis in 91U ECs using Annexin V and PI staining. (C) Quantification of EC apoptosis in vitro (n=5). (D) Immunofluorescent image from the in vitro study showing BCL2 (green) and Hoechst 33342 (blue). Scale Bar= 75 μm. Statistical significance: *p ≤ 0.05, ns is not significant.

**Figure S22.** *FOXF1* NP treatment increases BCL2 expression in pulmonary endothelial cells after injury. (A) Representative immunofluorescence images of lung sections stained for BCL2 (magenta) and CD31 (green) in uninjured, LPS-treated, and LPS + *FOXF1* NP-treated groups. Scale bars are 50 µm. (B) Quantification of the Mean Fluorescence Intensity (MFI) for BCL2 in CD31+ endothelial cells (n=3-6). Statistical significance: *p ≤ 0.05, **p ≤ 0.01.

**Table S1** **Antibodies and kits used for flow cytometry and immunostaining**

| Antibody | Company | Cat# |
| --- | --- | --- |
| CD326 | BioLegend | 118220 |
| CD31 | eBioscience | 48-0311-82 |
| CD140a | eBioscience | 25-1401-82 |
| CD45 | eBioscience | 56-0451-52 |
| CD117 | eBioscience | 78-1171-82 |
| NG2 | MilliporeSigma | AB5320C3 |
| FITC Annexin V Apoptosis Detection Kit | BD Bioscience | 556547 |
| CD31 (PECAM-1) | BD Biosciences | 553370 |
| Cdh5 (VE-cadherin) | Santa Cruz | sc-6458 |
| Clnd5 (Claudin-5) | ProteinTech | 29767-1-AP |
| Occludin | ProteinTech | 27260-1-AP |
| Bcl-2 mouse | ProteinTech | 26593-1-AP |
| BCL-2 human | Cell Signaling | 3498 |
| Cleaved PARP | Cell Signaling | 5625 |

**Table S2** **Primers for qRT-PCR analysis**

| SYBR green | Species | Seq |
| --- | --- | --- |
| *Gapdh* | *Mouse* | F:CATCACTGCCACCCAGAAGACTG  R:ATGCCAGTGAGCTTCCCGTTCAG |
| *Tjp1* | *Mouse* | F:GTTGGTACGGTGCCCTGAAAGA  R:GCTGACAGGTAGGACAGACGAT |
| *Ocln* | *Mouse* | F:TGGCAAGCGATCATACCCAGAG  R:CTGCCTGAAGTCATCCACACTC |
| *Cldn5* | *Mouse* | F:GGACACTGTCAGTCGTTGCAAC  R:ACAGTCGTAGGACTCGAACCAC |
| TaqMan | Species | Assay ID |
| *IL1b* | Mouse | Mm00434228_m1 |
| *IL6* | Mouse | Mm00446190_m1 |
| *TNFα* | Mouse | Mm00443258_m1 |
| *Pecam1* | Mouse | Mm01242576_m1 |
| *Cdh5* | Mouse | Mm00486938_m1 |
| *Foxf1* | Mouse | Mm00487497_m1 |
| *FOXF1* | Human | Hs00230962_m1 |
| *TEK* | Mouse | Mm00443243_m1 |
| *Flt1/VEGFR-1* | Mouse | Mm00438980_m1 |
| *TMEM100* | Mouse | Mm00471352_m1 |
| *Bcl2* | Mouse | Mm00477631_m1 |
| *Actb* | Mouse | Mm02619580_g1 |
| *ACTB* | Human | Hs01060665_g1 |
| *Ccl2* | Mouse | Mm00441242_m1 |
| *Cx3cl1* | Mouse | Mm00436454_m1 |
| *Vcam1* | Mouse | Mm01320970_m1 |
| *Egfr* | Mouse | Mm01187858_m1 |
| *Vegfa* | Mouse | Mm00437306_m1 |
| *Pdgfb* | Mouse | Mm00440677_m1 |
| *Wnt5a* | Mouse | Mm00437347_m1 |
